# The integration of health equity into policy to reduce disparities: Lessons from California during the COVID-19 pandemic

**DOI:** 10.1371/journal.pone.0316517

**Published:** 2025-03-06

**Authors:** Ada T. Kwan, Jason Vargo, Caroline Kurtz, Mayuri Panditrao, Christopher M. Hoover, Tomás M. León, David Rocha, William Wheeler, Seema Jain, Erica S. Pan, Priya B. Shete

**Affiliations:** 1 Division of Pulmonary and Critical Care Medicine, San Francisco General Hospital, University of California San Francisco, San Francisco, California, United States of America; 2 California Department of Public Health, Sacramento and Richmond, California, United States of America; 3 Division of Infectious Diseases, Department of Pediatrics, University of California San Francisco, San Francisco, California, United States of America; Universidad Nacional Autónoma de México Facultad de Medicina: Universidad Nacional Autonoma de Mexico Facultad de Medicina, MEXICO

## Abstract

Racial and ethnic minoritized groups and socioeconomically disadvantaged communities experience longstanding health-related disparities in the United States and were disproportionately affected throughout the COVID-19 pandemic. How departments of public health can explicitly address these disparities and their underlying determinants remains less understood. To inform future public health responses, this paper details how California strategically placed health equity at the core of its COVID-19 reopening and response policy, known as the *Blueprint for a Safer Economy*. In effect from August 2020 to June 2021, “the Blueprint” employed the use of the California Healthy Places Index (HPI), a place-based summary measure of 25 determinants of health constructed at the census tract level, to guide activities. Using California’s approach, we categorized the state population by HPI quartiles at the state and within-county levels (HPIQ1 representing the least advantaged, HPIQ4, the most advantaged) from HPI data available to demonstrate how the state monitored crude COVID-19 test, case, mortality, and vaccine rates and unadjusted rate ratios (RR) using equity metrics developed for the Blueprint. Notable patterns emerged. Testing disparities disappeared during the summer and winter surges but resurfaced between surges. Monthly case RR peaked in May 2020 for HPIQ1 compared to HPIQ4 (RR 6.61, 95%CI: 6.41–6.81), followed by mortality RR peaking in June 2020 (RR 5.06, 95% CI: 4.34–5.91). As the pandemic wore on, disparities in unadjusted case and mortality RRs between lower HPI quartiles relative to HPIQ4 reduced but remained. Utilizing a place-based index, such as HPI, enabled a data-driven approach that used a determinants of health lens to identify priority communities, allocate resources, and monitor outcomes based on need during a large-scale public health emergency.

## Introduction

Health disparities worsen during public health emergencies [1, 2], and the coronavirus disease 2019 (COVID-19) pandemic was no exception. It is well documented that racial/ethnic minoritized groups and socioeconomically disadvantaged communities in the United States (US) experienced higher incidence and severe health outcomes from COVID-19 [3–8]. Hospitalizations and deaths were reported to be 2 to 5 times higher among Black, Hispanic, and American Indian and Alaskan Native people compared to Asian and white counterparts [3–8]. Despite higher vaccination rates, people of color aged 55–64 experienced higher COVID-19 mortality than white people of the same age or even 10 years older during both Delta and Omicron surges [9]. Population groups experiencing these disparities and their underlying determinants have also experienced reductions in life expectancy [10], income loss, unemployment [11], and learning loss due to school closures [12] as a result of the pandemic or its mitigation strategies. However, even as evidence of health-related disparities continue to accrue, there are fewer documented instances in the US which describe how to effectively address underlying determinants and implement public health approaches that focus on mitigating health disparities.

A significant barrier to reducing disparities stems from methodological limitations in identifying populations that face inequities in an actionable, reproducible, and scalable manner [13–17]. Public health traditionally defines disparities using single factors, such as age, race/ethnicity, or income [18–22]. Using this information to address disparities in practice is often limited by: incomplete data, challenges in targeting interventions at the individual or household level, and not accounting for environmental and intersecting factors [20, 23, 24]. These approaches also fail to capture the complex interplay of multi-level factors often referred to as social determinants of health (SDOH), defined by the World Health Organization as “the conditions in which people are born, grow, live, work, and age, [including] the forces and systems shaping the conditions of daily life” [25].

Place-based approaches have emerged as promising strategies for identifying priority populations, defining disparities, and targeting resources in public health [26–28]. These approaches capture the specific geographic and social contexts of communities through measures that reflect location-specific characteristics, including demographics and SDOH [29–33]. While these approaches are commonly used in high-income settings (e.g., Canada, Great Britain, New Zealand) to guide health initiatives [34], their application in the US prior to the COVID-19 pandemic was mostly limited to disaster planning and preparedness [32]. During the pandemic, various place-based indices were used to describe disparities in COVID-19 incidence, hospitalizations, and mortality [35, 36]. However, their use in guiding interventions and programs has remained underdeveloped [31, 35].

Recognizing the need to address the harms disproportionately experienced by certain populations during the early stages of the pandemic and to prevent further widening of disparities and their onward consequences, the state of California implemented a pandemic response and reopening strategy with health equity at its core. According to the California Department of Public Health (CDPH), health equity refers to “efforts to ensure that all people have full and equal access to opportunities that enable them to lead healthy lives” and “requires a focus on SDOH–such as housing instability, food insecurity, social isolation, financial strain, and interpersonal violence—which are dominant causes of preventable disease and injury, and are often perpetuated by systemic racism” [37].

Health equity provided a framework for the state to decide how to allocate resources across its large geography and diverse population during a public health emergency. By using place-based health equity metrics based in SDOH and focused on neighborhood conditions, health equity was codified in California’s reopening and response policy the *Blueprint for a Safer Economy*. There is emerging evidence that California’s equity-focused approach, once it took effect, was successful at reducing COVID-19 related disparities, and promoting additional interest in health equity across the state [38]. However, a description of the state’s process of creating a framework that incorporates health equity has yet to be disseminated.

Building upon earlier publications on California’s equity-focused approach and policy [38–40], our objectives are to describe (i) how California explicitly integrated measures associated with health disparities into a public health response through the pandemic, both before and after vaccines became available, (ii) how the use of health equity metrics (i.e., approaches to quantify population-level outcomes with a health equity lens) aided in identifying and monitoring areas at-risk of suffering poor public health outcomes, and (iii) how these metrics offered a quantitative approach and a common language for shaping a public health response across a variety of stakeholders at the state, region, county, city, and community levels. We further demonstrate COVID-19 disparities in testing, incidence, mortality, and administered vaccine doses with the use of California’s COVID-19 equity metrics as a public health monitoring tool. Notably, we focus on providing an approach for operationalizing the use of equity metrics as a tool and for enhancing prioritization of public health resources with the goal of reducing health disparities actionably and at scale. For those interested in the causal effects of California’s approaches, which is outside the scope of this paper, we refer readers to the quasi-experimental and counterfactual analyses conducted by Hoover et al. [38].

## Materials and methods

### Study context

On August 30, 2020, the State of California launched “The Blueprint for a Safer Economy” (henceforth, “the Blueprint”), a statewide policy that focused on health equity for pandemic response, reopening, and monitoring strategies [41]. It was in effect until June 15, 2021, when the state fully reopened the economy. The Blueprint’s equity approach was both unique in the US and large in scale–with a population of 40 million people from diverse communities, California is the most populated US state, has the largest sub-national economy in the world, and contains a public health system encompassing 61 local health jurisdictions (LHJs; 58 counties and 3 cities) [10, 39].

The process to determine how to focus on health equity within the Blueprint was driven by political will and informed by data appropriateness, feasibility, and stakeholder engagement and after exploring a variety of approaches to identifying COVID-19 disparities. In June and July 2020, policy stakeholders and public health practitioners examined early COVID-19 testing, case, hospitalization, and mortality data with a variety of data sources, in order to identify individuals, groups, or places that may be “more vulnerable”, in line with how the state defined health equity and prioritized addressing SDOH [37]. Mobile phone data was assessed before and during the stay-at-home orders to identify whether mobility was associated with the different COVID-19 outcomes. Race and ethnicity indicators were utilized when available to assess potential associations between identifying as belonging to a minoritized community and COVID-19 outcomes. The state also analyzed COVID-19 outcomes by various area-based socioeconomic measures (ABSMs, also known as place-based indexes), which are typically composite measures that incorporate data on a community’s underlying SDOH at granular geographic levels, such as census tract or ZIP Code Tabulation Area [30, 42].

Ultimately, to identify populations for an equity-focused public health response, CDPH and partners elected to utilize a California-specific ABSM called the California Healthy Places Index (HPI) that quantitatively captured the “healthiness of community conditions” at the census tract level [29, 43]. Developed by the Public Health Alliance of Southern California (PHASC) who also advised on its incorporation into the Blueprint [44], HPI is a place-based composite index that aggregates 25 community characteristics, which are grouped into eight variable domains called “Policy Action areas” for a specific geographical area (e.g., census tract): economic, educational, social, transportation, healthcare access, neighborhood, housing, and clean environment (see **S1 Table** for Policy Action areas and their variables). With an extensive validation approach conducted in 2014, PHASC developed HPI based on literature, engagement with a steering committee with carefully selected expert members to representatively guide technical development for California, and a hybrid approach where indicators and Policy Action areas were selected based on actionability and empirical methods to optimize the association with a health outcome [29, 43]. This approach differs from other place-based indexes, which often use equal weighting for different indicators to construct a score that may not be tied to a health outcome [29, 43]. While not all census tracts have HPI scores (e.g., those with small populations where data is limited and the score cannot be constructed), tracts with a score were percentile ranked at the state *or* county level and divided into quartiles. **S1 Fig** depicts a map of census tracts by statewide HPI quartile. Details of the HPI, including development process, its construction, and validation, have been previously described, with the most relevant details included here [29, 43].

Among other options, such as other ABSMs or standard measures of inequality which have been empirically shown to reflect socioeconomic inequalities in health [31, 36, 45], HPI was determined as the most feasible for California’s pandemic policy by the state of California and CDPH for at least four main reasons:

■ Unlike other ABSMs, HPI was developed with a connection to a health outcome. HPI was constructed with Policy Action area z-scores that were calculated from the 25 constituent indicators at the census tract level and weighted using a quantile sums regression to maximize the overall index’s association with life expectancy at birth (LEB) in California [29]. HPI is positively correlated with LEB such that a unit increase in the HPI score correlates with a 3.5-year increase in LEB. Prior analyses have confirmed HPI’s accurate representation of SDOH in California’s most disadvantaged communities [29].
■ Since HPI was developed with LHJ, public health stakeholder, and community organization input across California, there was familiarity and existing buy-in with utilizing HPI at the time of the pandemic, as well as precedent for using it in other public health programming before the onset of the pandemic [46].
■ HPI’s asset-based framing, where higher HPI scores are interpreted positively, more as opportunities for living a healthy life, is unique. Many alternative indices highlight levels of vulnerability or disadvantage, which can further stigmatize communities.
■ HPI does not include race or ethnicity as constituent determinants, which while also a potential deficiency, in the context of California’s policy landscape was a benefit. California State Proposition 209 explicitly prohibits the use of race and ethnicity attributes to determine policies [25].

The Blueprint utilized both state and county HPI quartiles, where the 1^st^ quartile was interpreted as the 25% of the population having the least opportunity to live a healthy life; 4^th^, having the most opportunity [44]. HPI is also highly correlated with other ABSMs that are more well-known for use across US federal and other state and local health departments, such as the CDC’s Social Vulnerability Index (SVI), which was designed to help decision makers identify communities with the greatest or least vulnerability to environmental and public health hazards [44, 47]. Other studies have compared HPI with other measures, including SVI, CalEnviroScreen, poverty levels based on household incomes, the Intercity Hardship Index, the American Human Development Index, Home Owners’ Loan Corporation “redlining” scores, and more [29, 48–50].

**Fig 1:**
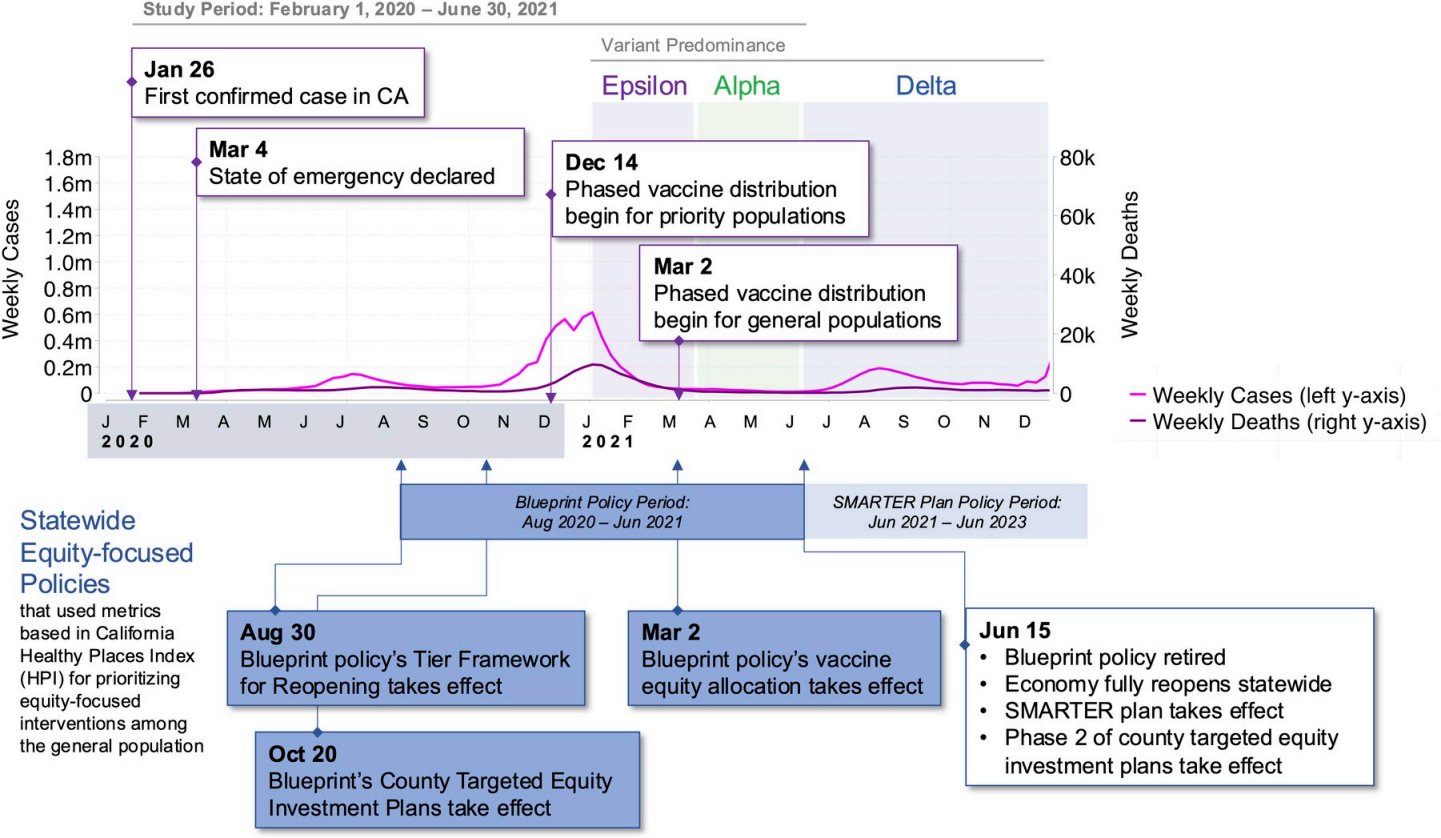
California timeline of COVID-19 pandemic including equity-focused policies. The Blueprint encompassed the winter surge at the end of 2020 and into 2021, when vaccines were phased in across priority populations and then later distributed to the general population. For vaccines, priority populations included persons at risk of exposure to SARS-CoV-2 through work in direct health care or long-term care settings; residents of skilled nursing facilities, assisted living facilities, and similar long-term care settings for older or medically vulnerable individuals. This was also a time when testing resources were no longer limited, as they had been earlier in 2020, when the first confirmed cases were identified and the months after the state of emergency was declared in California. Variants that were predominant during the Blueprint included Alpha and Epsilon, and the policy was retired before the Delta period. Note: COVID-19 time series and variant data from California Health and Human Services Open Data Portal.

The Blueprint included three equity-focused policy activities (underlined) that incorporated specific equity metrics adapted from HPI (***italicized and bolded***), summarized here and depicted in the **Fig 1** timeline:

1. The Tier Reopening Framework that took effect on August 31, 2020 established benchmarks for counties to meet in order to safely “reopen the economy” (i.e., liberalization of restrictions that allowed for re-opening of businesses, schools, and other activities). Weekly, California’s 58 counties were assigned one of four tiers that determined whether the county should increase, decrease, or maintain the same level of social and economic restrictions. Tier assignments were stratified by county size and transmission risk within a county, ascertained by test positivity and either case rate normalized to 100,000 residents or case counts, depending on county size.
  For county size, California designated counties with a 2019 county population greater than 106,000 as “large”, and counties with a population less than or equal to 106,000 as “small”. Classification by county size was adopted because California’s case rate metric, normalized per 100,000 residents, led to significant fluctuations in daily case rates in counties with small populations from a small number of newly reported cases. To avoid unnecessarily closing or restricting business sectors and other activities in small counties based on such variability from a small number of new cases, CDPH in collaboration with LHJs used absolute case counts (and later on, absolute vaccine doses) in counties with 106,000 residents or fewer. During the policy, 22 counties had a population less than 100,000, and an additional county which had a population of 106,000 was also included as a small county because it shared a health officer with another small county.
  Large counties had to meet equity-focused benchmarks to move towards reopening. Effective from October 6, 2020, the equity benchmark called the ***Health Equity Metric (HEM)*** was incorporated into the tier framework for large counties. Adapted from HPI, the HEM of a large county was the test positivity rate of the census tracts in the lowest within-county HPI quartile, referred to as ***the Health Equity Quartile (HEQ)***. The intention was to provide clear incentives for counties to prioritize equitable improvements in COVID-19 outcomes and prevent a sole focus on reducing the county’s overall transmission risk. Because of statistical challenges arising from a small number of census tracts in constructing a within-county HPI quartile, smaller counties were exempt from the ***HEM*** requirement for reopening [46]. Instead, rules for small counties adjusted over time, as CDPH worked together with these LHJs. Ultimately, small counties were assigned tiers based on meeting county-level case count and test positivity thresholds. Regions (designated by CDPH) and counties also were able to receive technical support from CDPH in meeting these metrics. Counties were also able to submit tier adjudication requests with supporting documentation in the case there were questions or considerations regarding data discrepancies or errors with weekly tier assignments.
2. County Targeted Equity Investment Plans were crafted by county (and later, city) LHJs to describe how resources would be mobilized to mitigate COVID-19 disparities within a jurisdiction. LHJs were encouraged to focus their plans on their ***HEQ*** or other disproportionately impacted communities based on their discretion. Plans were submitted to the state by October 20, 2020 and implemented over time. Based on these plans, counties initially committed approximately $280 million of federal funding, that was allocated to them, to disproportionately impacted communities [51].
3. The Vaccine Equity Allocation was later incorporated, taking effect on March 2, 2021, to guide vaccine allocation for the general population when vaccines became available. By this time, priority populations including older adults, people with certain comorbidities, and people living and working in settings with risks of exposure to SARS-CoV-2 (e.g., direct health care or long-term care settings, skilled nursing facilities, carceral settings), had already been offered vaccines in previous phases. In contrast with the Tier Reopening Framework and County Targeted Equity Investment Plans which both focused on the HEQ constructed at the county level (within-county HPI quartile 1), the ***Vaccine Equity Metric (VEM) Quartile*** was constructed at the statewide level and based on HPI scores assigned to ZIP Codes. The Blueprint’s Vaccine Equity Allocation sent 40% of the vaccine supply to ZIP Codes assigned to the VEM Quartile 1 (VEM Q1), where each of the other quartiles received 20% of the supply. As part of this Blueprint activity, two ***VEM*** goals were incorporated within the Tier Reopening Framework to reduce strictness of its thresholds, such that: (1) once two million vaccination doses had been administered in VEM quartile 1 (VEM Q1), the two most restrictive tiers would have less strict thresholds, and (2) once four million doses had been administered in VEM Q1, then the three least restrictive tiers would have less strict thresholds [52]. An evaluation of the impact of the vaccine equity allocation found that 160,892 COVID-19 cases, 10,248 hospitalizations, and 679 deaths were averted in the VEM Q1 during the eight-month period after the policy was implemented [38].

All three of these activities included technical assistance provided to LHJs by the state. For additional Blueprint details, including tier thresholds by county size, see **S1 Text**.

### Data and outcomes

We constructed a time-series dataset to depict COVID-19 health outcomes across statewide and within-county HPI quartiles using data from February 1, 2020 to June 30, 2021. This period encompasses the beginning of COVID-19 transmission in California, the implementation of the Blueprint’s Tier Reopening Framework and targeted equity investment plans, the summer 2020 surge, and a larger surge during the winter of 2020–2021, the introduction of vaccines among the general population, and the full reopening of the economy, coinciding with the retiring of the Blueprint policy (**Fig 1**). We did not include periods of Delta or Omicron variant predominance. We focused on COVID-19 health outcomes related to testing, cases, and COVID-19 associated mortality, as well as vaccine doses administered. Estimation of the effects of the Blueprint’s Vaccine Equity Allocation on COVID-19 outcome were reported previously [38].

Data in this study included geocoded CDPH COVID-19 surveillance and vaccination data, COVID-19 time series and variant data from the California Health and Human Services Open Data Portal [53–55], American Community Survey (ACS) 2019 5-year estimates for population and population share comprised of race and ethnicity groups [56], and HPI data from PHASC at the census tract level [43, 57]. The surveillance data were person-level records of confirmed SARS-CoV-2 infection in California, which were collapsed to weekly COVID-19 tests, positive tests, cases, hospitalizations, and deaths. Since the Blueprint policy was implemented for the general California state population, we excluded: (a) persons out of state or with unknown county of residence, and (b) persons incarcerated at state or federal prisons, Immigration and Customs Enforcement facilities, US Marshall only detention facilities, and Department of State hospitals. Population-normalized rates per 100,000 were computed for tests, cases, deaths, and vaccine doses administered using 2019 ACS 5-year population estimates.

Following California’s policy approach, we presented outcomes that were actionable for surveillance, policy rules, and ongoing monitoring by the state, LHJs, and community organizations throughout the pandemic. Specifically, these include crude test, case, and mortality rates (following the Tier Reopening Framework); test positivity (following the Tier Reopening Framework); and total number of vaccine doses administered (following the Tier Reopening Framework after vaccines were introduced and Vaccine Equity Allocation). Definitions for COVID-19 tests, confirmed cases, and COVID-19 deaths were reported and defined by CDPH, following definitions set by the Council of State and Territorial Epidemiologists and published by the CDC [58]. Tests are the total number of SARS-CoV-2 molecular tests conducted (nucleic acid amplification tests, including polymerase chain reaction tests), based on specimen collection date to account for any reporting delays. Test positivity for a given period is the percentage of positive SARS-CoV-2 tests over the number of tests, as reported to CDPH by performing laboratories and health systems. Testing data included addresses and demographic characteristics, such as race and ethnicity, self-reported by individuals tested. Cases are individuals with a laboratory-confirmed, positive SARS-CoV-2 test as reported to the CDPH. COVID-19 deaths refer to individuals with confirmed COVID-19 associated deaths reported to the CDPH by LHJs. Vaccines administered are the number of doses administered by ZIP Code. To ensure no areas were excluded from the vaccine allocation, additional imputation was conducted for CDPH-derived ZIP Code Tabulation Areas that were originally excluded from the original HPI construction due to data sparsity and statistical reliability. Since the first vaccine in California was administered on December 14, 2020, we report the outcome for vaccine doses administered for the period December 1, 2020 to June 30, 2021.

For non-vaccine outcomes, census tracts had been assigned by CDPH to an individual’s residence as per addresses reported to California Electronic Lab Reporting. Residential addresses that were listed with COVID-19 data had been geocoded by CDPH using the ArcGIS StreetMap Premium geocoding service (Environmental Systems Research Institute, Redlands, California, US). Geocoded cases were categorized by tracts defined by the US Census Bureau 2010 decennial census. Cases and tests without a geocoded latitude and longitude and those within 50 meters of a prison or registered skilled nursing facility were excluded from the analysis.

### Analytic approach

We computed descriptive statistics for census tracts in the state, among large counties and small counties, as well as for all statewide HPI quartiles pooled and by each quartile. County size (i.e., small, large) was classified according to the Blueprint’s criteria. We computed unadjusted rate ratios (RR) for cases and mortality with 95% confidence intervals (CIs) at the tract level and reported results for HPI quartiles 1 (HPIQ1), 2 (HPIQ2), and 3 (HPIQ3) with quartile 4 (HPIQ4) as the reference group for the entire study period and by month. To denote outcome disparities, RRs for HPIQ1 relative to HPIQ4 was referred to as “HPIQ1:Q4”; for HPIQ2, “HPIQ2:Q4”; for HPIQ3, “HPIQ3:Q4”. Crude weekly outcomes for test, case, and mortality rates are depicted as absolute numbers and proportions by HPI quartiles and by race groups that were self-reported at point of testing. We reported weekly rates for vaccine doses administered (per 100,000) by VEM quartiles for the period December 1, 2020 to June 30, 2021.

### Ethical clearance

Analyses conducted were considered not research or exempt from the State of California Health and Human Services Agency’s Committee for the Protection of Human Subjects (Project #2023–193), as data and results were considered essential components of California Department of Public Health public health surveillance.

## Results

Across California in 2019, there were 58 counties, 8,057 census tracts, and a population of approximately 39.3 million (**Table 1**). Based on the Blueprint’s definition of county sizes, there were 35 large counties (i.e., with more than 106,000 population), that made up 97.3% (7,839) of the census tracts in the state and 97.6% of the state’s population. The remaining 23 small counties in California were comprised of 218 (2.7%) census tracts and 2.4% of the state population. **Table 2** contains summary statistics of census tracts across California, large counties, and small counties (where data existed) for demographic characteristics and variables within the eight HPI Policy Action areas: economic, educational, social, transportation, healthcare access, neighborhood, housing, and clean environment.

**Table 1:** Summary statistics. California counties and census tracts by county size categories.

County size	Number of Counties	Number of CTs	Population
	(% of total)	(% of total)	(% of total)
**All Counties**	58	8,057	39,283,497
	(100.0)	(100.0)	(100.0)
**Large Counties**	35	7,839	38,352,825
	(60.3)	(97.3)	(97.6)
**Small Counties**	23	218	930,672
	(39.7)	(2.7)	(2.4)

Note: County and census tracts are based on the 2010 geographic areas from the U.S. census bureau. Population is from American Community Survey 2015–2019 5-year estimates. Large counties are counties with a 2019 population of greater than 106,000.

**Table 2:** Summary statistics. Summary statistics for census tracts in California, large counties, and small counties: characteristics across demographics and the eight HPI policy action areas (economic, educational, social, transportation, healthcare access, neighborhood, housing, and clean environment).

	Tracts in California (N = 8,057)		Census Tracts in Large Counties (N = 7,839)		Census Tracts in Small Counties (N = 218)	
	N	Mean	N	Mean	N	Mean
Population (2019)	7,793	4,977.52	7,589	4,992.11	204	4,434.78
Life expectancy at birth	7,767	80.27	7,568	80.32	199	78.67
Proportion 65 years or older	7,793	0.13	7,589	0.13	204	0.19
Proportion White	7,793	0.39	7,589	0.37	204	0.68
Proportion Black	7,793	0.06	7,589	0.06	204	0.01
Proportion Asian	7,793	0.14	7,589	0.14	204	0.03
Proportion Native American	7,793	<0.01	7,589	<0.01	204	0.02
Proportion Pacific Islander	7,793	<0.01	7,589	<0.01	204	<0.01
Proportion Other Race	7,793	<0.01	7,589	<0.01	204	<0.01
Proportion Multiple Races	7,793	0.03	7,589	0.03	204	0.03
Proportion Latino/a	7,793	0.39	7,589	0.39	204	0.21
Healthy Places Index v2.0	7,793	50.01	7,589	50.24	204	41.25
**Economic**						
Proportion with an income exceeding 200% of federal poverty level	7,767	0.69	7,568	0.69	199	0.64
Proportion aged 25–64 who are employed	7,767	0.72	7,568	0.72	199	0.64
Per capita income	7,767	38,056.13	7,568	38,262.21	199	30,218.70
**Education**						
Proportion over age 25 with a bachelor’s education or higher	7,767	0.33	7,568	0.33	199	0.21
Proportion of 15-17-year-olds enrolled in school	7,767	0.98	7,568	0.98	199	0.97
Proportion of 3- and 4-year-olds enrolled in pre-school	7,767	0.53	7,568	0.53	199	0.49
**Social**						
Proportion of households who completed census forms (2020)	7,767	0.70	7,568	0.70	199	0.59
Proportion of registered voters voting in the 2020 general election	7,767	0.78	7,568	0.78	199	0.80
**Transportation**						
Proportion of households with access to an automobile	7,767	0.93	7,568	0.93	199	0.95
Proportion of workers (16 years and older) commuting by walking, cycling, or transit (excluding working from home)	7,767	0.09	7,568	0.09	199	0.05
**Healthcare Access**						
Proportion of adults aged 18 to 64 years currently insured	7,767	0.89	7,568	0.89	199	0.89
**Neighborhood**						
Proportion living within ½ -mile of a park, beach, or open space >1 acre	7,767	0.77	7,568	0.78	199	0.52
Population-weighted percentage of the census tract area with tree canopy	7,767	0.08	7,568	0.07	199	0.23
Employment density (jobs/acre)	7,767	6.97	7,568	7.11	199	1.28
**Housing**						
Proportion of occupied housing units occupied by property owners	7,767	0.55	7,568	0.55	199	0.66
Proportion of households with complete kitchen facilities and plumbing	7,767	0.99	7,568	0.99	199	0.99
Proportion of low-income homeowners paying >50% income on housing	7,767	0.12	7,568	0.12	199	0.10
Proportion of low-income renter households paying >50% income on housing	7,767	0.25	7,568	0.25	199	0.23
Proportion of households with less or equal to 1 occupant per room	7,767	0.91	7,568	0.91	199	0.96
**Clean Environment**						
Annual average spatial distribution of gridded diesel PM emissions from on-road and non-road sources 2016 (tons/year)	7,767	0.22	7,568	0.23	199	0.05
Drinking water contaminant index for selected contaminants (CalEnviroScreen 4.0)	7,767	477.36	7,568	478.86	199	420.38
Mean of summer months (May-October) of the daily max 8-hour ozone concentration (ppm), averaged over 2017 to 2019	7,767	0.05	7,568	0.05	199	0.05
Annual mean concentration of PM2.5 (μg/m3) over three years (2015–17)	7,767	10.18	7,568	10.28	199	6.51

Note: HPI is California Healthy Places Index, version 2.0. HEQ is the “health equity quartile” or within-county HPI quartile 1; Non-HEQ refers to the within-county HPI quartiles 2, 3, and 4. Population; proportions by age, race, and ethnicity; life expectancy; all economic, education, transportation, and healthcare access measures are from ACS 2015–2019 5-year estimates. Registered voters is from UC Berkeley 2020. Census response is from Decennial census 2020. Neighborhood measures are from Greeninfo (2012), NLCD (2011), and USEPA (2010), respectively. Housing measures are from ACS 2015–2019 5-year estimates or CHAS 2010–2014. Clean environment variables are from CalEPA (2016), CalEPA (2011–2019), CalEPA (2017–2019), and CalEPA (2015–2017), respectively. HPI v2 source: https://phasocal.org/wp-content/uploads/2018/04/HPI2Documentation2018-04-04-FINAL.pdf. HPI v3 source: https://assets.website-files.com/613a633a3add5db901277f96/63320a9e98493bbdcc03d509_HPI3TechnicalReport2022-09-20.pdf.

Overall, 7,793 (96.7%) of California’s tracts in 57 counties had HPI scores with 1,948 tracts (24.2%) in HPIQ4, HPIQ3, and HPIQ2 each and 1,949 tracts (24.2%) in HPIQ1 (**Table 3**). Tracts with an HPI score had a total population of approximately 38.8 million, or 98.7% of the state’s 2019 population. HPI quartile populations ranged from 9.42 million to 9.94 million residents, corresponding to 24.0% to 25.3% of the total population, respectively. When the state constructed VEM quartiles based on ZIP Codes, 99.8% of California’s population fell within any VEM quartile, with 27.0% of the population in VEM Q1, 25.2% in VEM Q2, 23.9% in VEM Q3, and 23.7% in VEM Q4 (**Table 4**). **S2 Table** reports similar statistics as Tables 1 and 2 for tracts in California, large counties, and small counties by HEQ (within-county quartile 1) or non-HEQ quartiles (within-county quartiles 2–4). **S3 Table** reports statistics by county population categories and county.

**Table 3:** Summaries and cumulative COVID-19 metrics by statewide HPI quartile from Feb 1, 2020 through Jun 30, 2021. By statewide HPI quartiles.

HPI Quartile	Number of Counties	Number of Census Tracts	Population	Tests conducted	Cases reported	Deaths reported
	(% of total)	(% of total)	(% of total)	(% positive)	(per 100k)	(per 100k)
All quartiles pooled	57	7,793	38,789,824	37,885,618	3,143,037	54,006
	(98.3)	(96.7)	(98.7)	(10.4)	(8,102.7)	(139.2)
HPI Q4	35	1,948	9,575,270	9,296,662	369,217	6,329
(Most Opportunity)	(60.3)	(24.2)	(24.4)	(4.5)	(3,855.9)	(66.1)
HPI Q3	53	1,948	9,944,751	9,584,731	665,355	11,664
	(91.4)	(24.2)	(25.3)	(7.9)	(6,690.5)	(117.3)
HPI Q2	55	1,948	9,850,779	9,728,422	958,483	16,224
	(94.8)	(24.2)	(25.1)	(11.4)	(9,730.0)	(164.7)
HPI Q1	43	1,949	9,419,024	9,275,803	1,149,982	19,789
(Least Opportunity)	(74.1)	(24.2)	(24.0)	(14.7)	(12,209.1)	(210.1)

**Table 4:** Summaries and cumulative COVID-19 metrics by statewide HPI quartile from Feb 1, 2020 through Jun 30, 2021. By statewide vaccine equity metric (VEM) quartiles.

VEM Quartile	Population	Vaccine Doses Administered
	(% of total)	(per 100k)
All VEM quartiles pooled	39,216,122	41,628,117
	(99.8)	(106,150.5)
VEM Q4	9,298,697	12,421,452
(Most Opportunity)	(23.7)	(133,582.7)
VEM Q3	9,397,006	10,477,969
	(23.9)	(111,503.3)
VEM Q2	9,902,776	9,976,732
	(25.2)	(100,746.8)
VEM Q1	10,617,643	9,049,444
(Least Opportunity)	(27.0)	(85,230.3)

Note: HPI is California Healthy Places Index (HPI). Census tracts were percentile ranked by their HPI scores at the state level and divided into quartiles. “Q4” is interpreted as the 25% of California’s population that has the most opportunity to live a healthy life, whereas Q1 is the 25% with the least opportunity to live a healthy life. Census tracts are based on 2010 census. Population is based on 2019 ACS 5-year estimates. Vaccine equity metric (VEM) quartiles (Q1, Q2, Q3, Q4) are based on HPI scores at the ZIP Code level and cover a larger population than HPI quartiles because additional imputation was conducted for ZIP Code Tabulation Areas that were originally excluded from HPI construction due to data sparsity and statistical reliability.

We analyzed COVID-19 case data using HPI as compared to race indicators from the COVID-19 surveillance data. We found that only a small proportion of cases could not be classified with HPI, whereas using race indicators resulted in between 50–75% of cases as unclassifiable at any given time (see **S2 Fig, panels a and b**). Similar findings were noted in hospitalization and mortality data from case surveillance data (**S2 Fig, panels c-f**). To assess the association between different race/ethnicity groups and HPI, we examined the percentage of race/ethnicity groups residing in census tracts across HPI percentiles (**S3 Fig**). Individuals from historically minoritized communities, such as Latino/a, Black, American Indian/Alaska Native, and Native Hawaiian/Other Pacific Islander populations, make up a plurality within tracts with the least opportunity to live healthy lives.

### Testing

Between February 2020 and June 2021, approximately 37.89 million tests were conducted of which the most tests were conducted among residents of HPIQ2 (9,728,422, 25.7% of all tests; **Table 3**). The fewest tests were conducted among residents of HPIQ1 (9,275,803, 24.5%). Testing rates ranged from 96,872/100,000 in HPIQ1 to 101,599/100,000 in HPIQ2, with HPIQ3 and HPIQ4 with rates of 100,099/100,000 and 97,090/100,000, respectively.

### Cases

In the study period, 3,143,037 cases were reported across all HPI quartiles, which resulted in a cumulative case rate of 8,103/100,000 (**Table 3**). HPIQ4 had 369,217 cases with total cases increasing with decreasing opportunity. HPIQ1 had the most cases (1,149,982). Adjusting for population has a similar pattern. Cumulative case rate was the lowest for HPIQ4 (3,856/100,000) and then increased with decreasing opportunity with the highest observed in HPIQ1 (12,209/100,000). The state’s overall test positivity rate was 10.4% for the 17-month period examined. Test positivity rates were lowest in HPIQ4 (4.5%) and highest in HPIQ1 (14.7%).

**Fig 2:**
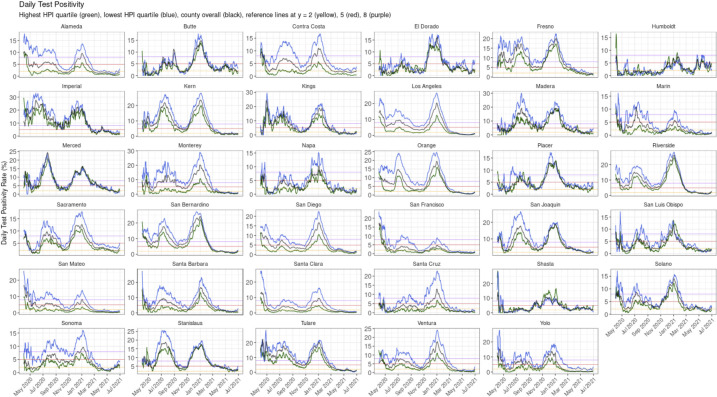
Daily test positivity rate (%) of COVID-19 for California’s 35 large counties from Apr 1, 2020 to Jun 30, 2021, where the lowest HPI quartile test positivity (blue) is the daily health equity metric used in the Blueprint. Note: Large counties are defined as those with a 2019 ACS population greater than 100,000. Daily test positivity is a percentage of daily new positives over total tests conducted that day. HPI quartiles displayed for each county are assigned at the state level. Dark and light blue lines trace the daily test positivity of a county’s HPI quartile 1 and 2 census tracts, respectively. Light and dark green lines trace the daily test positivity of the county’s HPI quartile 3 and 4 census tracts, respectively.

We further analyzed daily test positivity in accordance with California’s implementation of the HEM, which measured test positivity by county-level HPI quartiles for the 35 large counties (**Fig 2)**. Results showed higher test positivity rates in the HEQ (within-county HPI quartile 1, depicted in blue) across counties and over time, particularly in the period before the end of the winter 2020–21 surge (Nov 2020 –Jan 2021). Across the counties after the surge through the end of the study period, we generally see two patterns that coincide with each other: a reduction in overall test positivity and a reduced disparity in test positivity between the highest within-county HPI quartile (depicted in green) and the HEQ (depicted in blue).

### Deaths

The relationship between HPI quartiles and cases was similar for COVID-19 mortality. For all quartiles, 54,006 deaths were reported at a rate of 139/100,000 (**Table 3**). The fewest deaths occurred in HPIQ4 (6,329) with the number of deaths increasing with decreasing opportunity. Total deaths increased to 11,664 in HPIQ3 and then to 16,224 in HPIQ2. Among residents in HPIQ1 communities, a total of 19,789 COVID-19 deaths occurred. Cumulative mortality rate was lowest for HPIQ4 (66/100,000) and decreased with increasing opportunity with the highest observed for HPIQ1 (210/100,000).

### Vaccines

Between December 1, 2020 to June 30, 2021, over 41.6 million vaccine doses were administered across California, at a rate of 106,150.5 per 100,000 population. In the study period, over 9 million doses were administered among VEM Q1. We observe that the highest rate of vaccines administered was in VEM Q4 (133,582.7/100,000) with decreasing rates with decreasing opportunity to live a healthy life. VEM Q1 had the lowest rate of vaccines administered at 85,230.3/100,000.

### Time trend analysis

**Fig 3:**
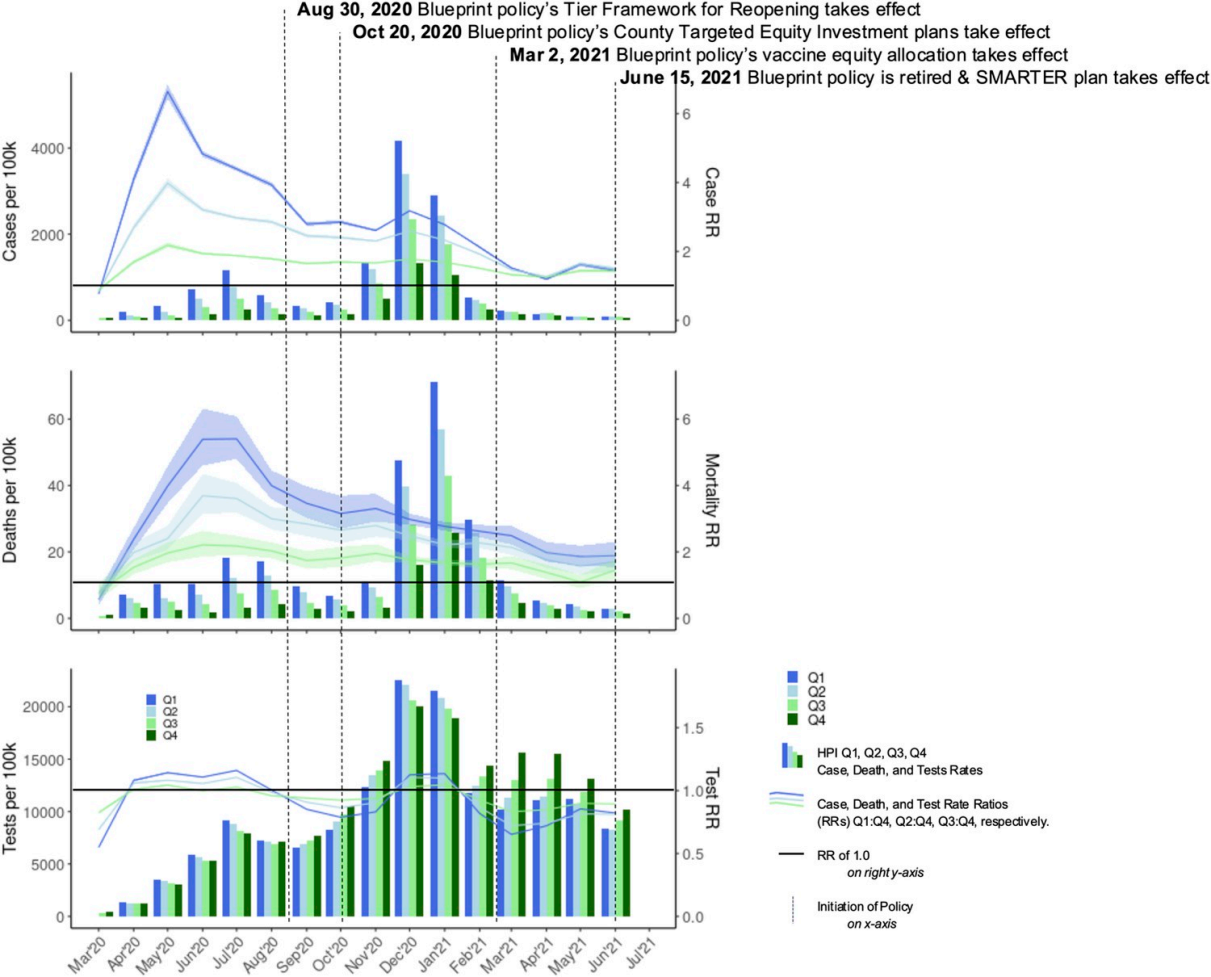
Monthly rates per 100,000 population and unadjusted rate ratios by HPI quartiles for crude case, mortality, and testing rates. Note: Bars represent the rate per 100,000 population stratified by HPI quartile (left axis). Lines represent the unadjusted rate ratio with Q4 as the reference group (right axis) where a rate ratio of 1.0 is designated with the horizontal dashed line. Vertical dotted lines represent the onset of Blueprint policy’s tier framework for reopening (August 30, 2020) and the county targeted equity investment plans (October 20, 2020). Figure excludes February 2020 for graphing purposes; data corresponding to lines are shown for entire 12-month period in S4 Table.

We first compared crude monthly testing, case, and mortality rates across HPI quartiles (**Fig 3**, bar graph, left axis). Trends were similar to those seen in the cumulative analysis. Between March 2020 and January 2021, crude case rates were significantly higher in HPIQ1 as compared to all other quartiles for each month except in March 2020 (**Fig 3**, top). This corresponds to when unadjusted testing RR was highest for HPIQ3:Q4 and lowest for HPIQ1:Q4 (**Fig 3**, bottom, line graphs, right axis). Crude mortality rates in HPIQ1 were highest compared with all other quartiles for each calendar month since the onset of the pandemic in California except for March 2020 (**Fig 3**, middle). Notably, unadjusted monthly case RR for HPIQ1:Q4 peaked at 6.61 in May 2020 with the unadjusted monthly mortality RR peaking at 5.06 a month later (**S4 Table** for results with CIs), consistent with average mortality lagging infection by 2–3 weeks in the COVID-19 literature.

Between February and June 2021, the disparities in crude case and mortality rates among quartiles reduced, corresponding with reduced levels of infection after the winter 2020–21 surge. For testing, we observed significant disparities corresponding to lower testing rates in lower quartiles relative to HPIQ4 during February and March 2020. Statistically significant disparities in unadjusted testing RRs among lower quartiles relative to HPIQ4 disappeared in the months from April to July 2020, only to reappear again in August-November 2020 (**S4 Table** for results with CIs). After the winter 2020–21 surge, HPIQ4 maintained the highest monthly test rate through the end of the study period.

**Fig 4:**
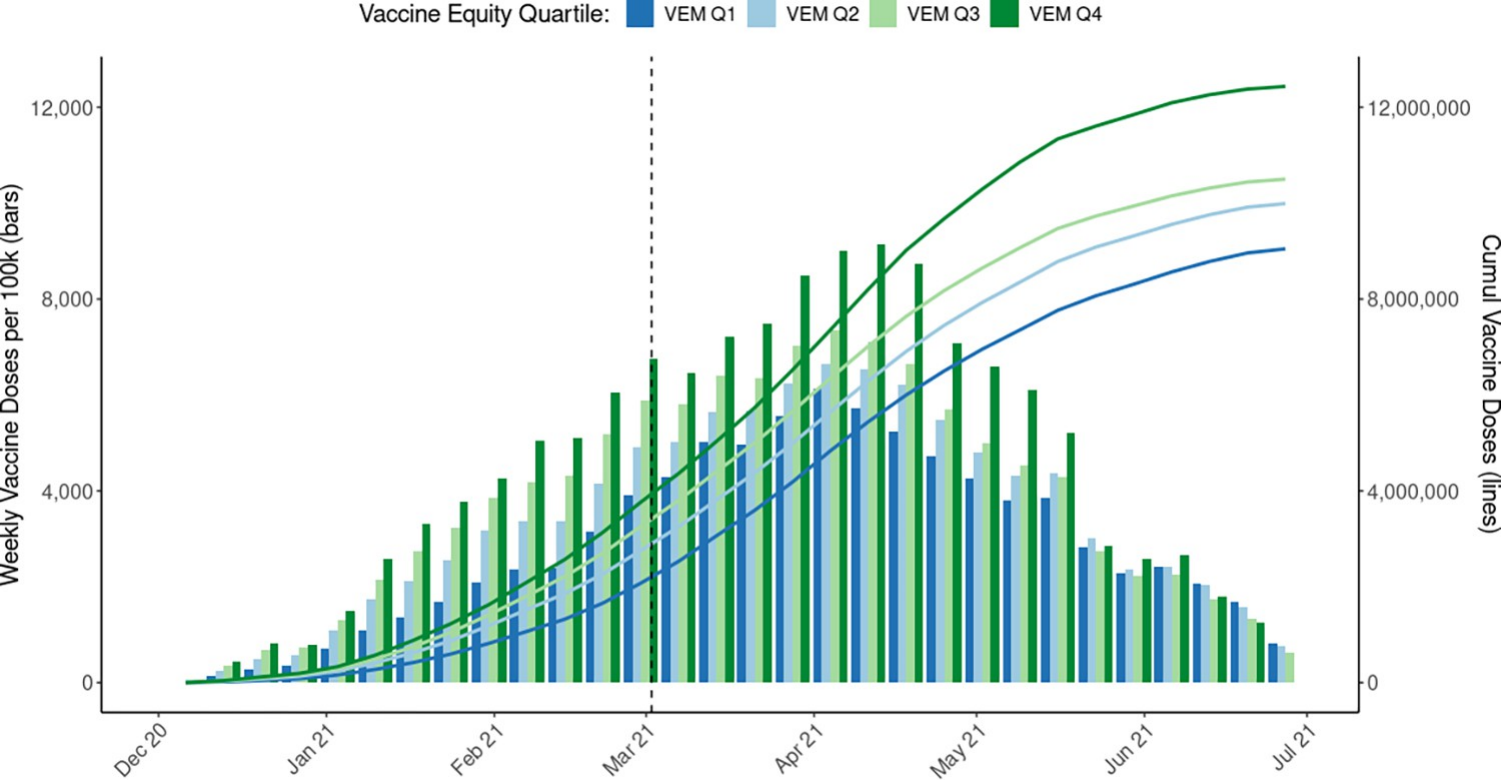
Crude weekly vaccination doses administered per 100,000 population (bars) and cumulative vaccine doses (lines) by Vaccine Equity Metric quartiles. Note. Vaccine equity metric quartiles (VEM Q1-Q4) are based on California Health Places Index (HPI) scores at the ZIP Code level and cover a larger population than HPI quartiles because additional imputation was conducted for ZIP Code Tabulation Areas that were originally excluded from HPI construction due to statistical reliability. Population is based on 2019 ACS 5-year estimates.

In **Fig 4**, the number of doses per 100,000 population administered by VEM quartiles corresponded to the opportunity to live a healthy life with disparity between VEM Q4 and VEM Q1 until March 2021, when it began to decrease. Towards the end of March into April, the greatest number of weekly doses were administered statewide, and thereafter, the disparities in crude rates of weekly vaccine doses among VEM quartiles began to converge through the end of June 2021. However, VEM Q4 consistently had the highest weekly vaccine dose rates compared to other quartiles until June 2021, when, for the first time, monthly vaccine doses per 100,000 population in VEM Q1 was the highest, and in VEM Q4, the lowest. In VEM Q1, the cumulative number of vaccine doses surpassed both 2 million in early March 2021 and then 4 million later the same month.

## Discussion

Our results demonstrate that using an ABSM such as HPI to describe epidemiologic trends, define disparities, monitor inequity, and guide policies was feasible at large scale within the context of emergency preparedness and response, resource mobilization, and public health initiatives. Our results indicate that Californian geographical areas with less opportunity for living a healthy life, as described by the HPI and not adjusting for any other factors, also had greater risk of infection and death related to COVID-19. Hence, using this ABSM for actionable policy had the advantage of allowing for a standardized and multidimensional assessment of the cumulative risk associated with the intersectionality of SDOH in California’s pandemic response. This type of comprehensive framing improved the understanding of influential determinants of disease for the state, suggesting that multidimensional metrics are essential to more accurately tracking disparities rather than relying on one measure alone [59]. Utilizing measures that map to specific social determinants at a large scale also supported the development of strategies and the use of similar language to overcome these barriers, as in California’s use of HPI-informed metrics to deploy resources towards equity programming within LHJs based on need. Agencies interested in similar approaches for public health initiatives may want to explore which measures (e.g., ABSMs and/or individual-level measures) are most appropriate and actionable for conducting surveillance, targeting interventions, allocating resources, and monitoring outcomes in their settings.

Examining COVID-19 data based on HPI quartiles revealed notable disparities in the epidemiology of disease burden, while complying with California’s Proposition 209. Census tracts in HPIQ1 had higher crude case and mortality rates than other communities statewide, apart from February and March 2020, when unadjusted outcome RRs for HPIQ1 and HPIQ2 communities were significantly lower than 1.0, reflecting overall low case counts and limited testing availability. Over time, despite increases in testing capacity, communities in HPIQ1 had disproportionately low testing rates compared to other quartiles, except during “surge” months when cases were highest. This finding has at least two implications. First, analyzing cumulative testing rates among HPI quartiles can mask disparities in testing which are more apparent when reviewed over time. Second, the uptake of testing only during times of higher need can highlight challenges to regular asymptomatic testing access for HPIQ1 during times of relative “calm”. Addressing testing disparities is vital for mitigating health inequities and could facilitate the identification of individuals needing quarantine in communities with elevated risks of adverse health outcomes.

Beyond descriptive statistics, standardizing an equity approach based in SDOH across California enabled the development of metrics based on need that could be actionably incorporated into policy and public health surveillance. While there were advantages and potential drawbacks [39], the development and inclusion of HPI in the Blueprint marks the purposeful integration of a health equity measure at scale to identify disproportionately impacted communities leading to deliberate prioritization of prevention and mitigation efforts to reduce health-related disparities [59]. Our analysis revealed that communities with the least opportunity suffered by up to six times the COVID-19 infection burden and up to 5 times the mortality burden during the summer of 2020, before the Blueprint took effect.

After the Blueprint took effect, statewide disparities across HPI quartiles persisted despite decreases in unadjusted case and mortality RRs, when compared to the group with the most opportunity. While case RR discrepancies between HPIQ1:Q4 and HPIQ2:Q4 diminished over time, the gap between HPIQ2:Q4 and HPIQ3:Q4 remained constant. This pattern held similarly for mortality. Notably, the winter surge had higher crude case and mortality rates than the summer surge but did not reach the same level of disparities as measured through unadjusted RRs. For instance, monthly HPIQ1:Q4 case RR was 3.16 in December and 2.79 in January; the HPIQ1:Q4 mortality RR in December was 3.03 and 2.77 in January (**S4 Table**).

When analyzing test positivity data as shown in **Fig 2**, we found higher test positivity rates in within-county HPIQ1 across counties and over time. HEM likely supported simultaneous monitoring of multiple dimensions of COVID-19, providing actionable information to large county LHJs. For example, in settings in which community testing rates remained low in spite of high test positivity, increases in testing were warranted. In settings in which test positivity remained high, despite concomitant high rates of testing, additional interventions were implemented to mitigate community virus spread. Contextual factors also likely influenced the manner in which efforts were implemented, which is an area of how to reduce disparities and operationalize health equity that warrants future research.

Regarding vaccines, we presented crude weekly vaccine dose rates and cumulative vaccine doses by VEM quartiles to show the vaccine equity measures included in the Blueprint, including those used in the Tier Reopening Framework. Our analysis did not account for the complex phasing of vaccinations across the state that began in December 2020 (e.g., people at risk of exposure from their role in health care or long-term care settings, residents of skilled nursing facilities or prisons). Because of this, we interpreted dose rates across the study period with caution beyond the thresholds for the Blueprint. For those interested in a causal analysis of California’s approach, recent evidence has demonstrated that the vaccine equity allocation averted COVID-19 health outcomes for VEM Q1 and that these results were robust to sensitivity analyses that tested exchangeability between communities prioritized in HPIQ1 versus those not prioritized by the allocation policy, different model specifications, and potential temporal confounders, including natural immunity [38]. Future research should also investigate the extent the Blueprint’s nonpharmaceutical efforts, prioritizing HPIQ1, causally contributed to the observed trends in reduced disparities in case and mortality rates across HPI quartiles after the winter 2020–2021 surge, as well as the extent disparities were reduced by other factors, such as natural immunity, herd immunity from high rates of prior infections in the communities, and survivorship bias.

In addition to contributing to the evidence on integrating equity metrics for public health initiatives, California’s COVID-19 policy approach offers several policy implications for agencies interested in utilizing a place-based SDOH framework within public health efforts aimed at reducing disparities at a large scale. Developed in response to a need expressed by state and local health departments and community organizations for an index that reflected SDOH that could help target place-based policies and programs, California’s HPI provided a geographically detailed, actionable policy-linked tool validated against LEB [29, 43]. It was constructed with input from a steering committee that included epidemiologists, policy leaders, and community organization representatives and was led by PHASC, a coalition of eight local health departments in California responsible for half of California’s state population [29, 43]. In this sense, agencies should carefully evaluate which measures best reflect the goals, needs, and local conditions by not only drawing from quantitative and qualitative evidence, but also community engagement and participatory approaches that involve key groups and individuals who are experts in local dynamics. For example, in addition to individual or household level variables, health departments can benefit from more geographically detailed and place-based measures, as well as direct engagement with communities, to more actionably identify areas of need and to gain support for ensuring targeted interventions can reach communities who may benefit most [60]. While quantitative data can be helpful, they should not be used alone to identify needs of communities [60].

Additionally, when using place-based measures, agencies may be concerned about data being out of date or not updated frequently enough for policy use and implementation. The version of HPI (2.0) used for California’s pandemic policy was published in 2015 and was primarily constructed from data sources dated between 2010 and 2015 (see full list in **S1 Table**) [29, 43]. At the onset of the pandemic, HPI was considered sufficient to reflect the living conditions statewide for policy. Since the integration of HPI version 2.0 in California’s COVID-19 policy, PHASC released version 3.0 in 2022 [61]. Since HPI itself does not represent longitudinal changes across the state, it is important to note that neighborhood and policy changes rarely occur over the course of 6–12 months, and there are few differences between HPI versions statewide.

The Blueprint approach itself encountered major limitations. First, generating county-level estimates of HPI-based outcome comparisons proved challenging for 23 of California’s 58 counties due to their small population sizes (**S2 Table**). These “small counties” had a total population of less than 1 million (2.4% of California’s population) and required different metrics from large counties (e.g., case count instead of case rate in the state’s Tier Reopening Framework) to mitigate small number issues for adhering to the Blueprint (e.g., a single COVID-19 case resulting in a large swing in the county case rate). Similar issues among small population areas have arisen when developing hospitalization forecasting models [62]. Alternative approaches should be developed to account approaches to more readily identify disparities in rural communities, as well as for rarer outcomes of interest.

Second, the Blueprint’s framework did not explicitly include structural racism as a determinant nor did it directly include race or ethnicity because of Proposition 209 [63]. However, studies have concluded the need to incorporate structural racism measures as it has been shown to influence the distribution of and access to resources and opportunities for and beyond health [50]. Addressing underlying determinants that perpetuate disparities may help link positive policy solutions, while avoiding the potential pitfalls of solely targeting interventions based on race/ethnicity [64]. Our analysis demonstrated a strong correlation between geography and the plurality of minoritized groups. Additional community engagement research can help better understand the limitations of these approaches to quantifying disparities more clearly. Additionally, further research is needed to evaluate the relative utility of different approaches, ABSMs, indices, and their constituents for equity-related policies, such as expanding resources to communities who receive fewer resources and experience the greatest disparities, stress due to worse health, as well as the consequences of accumulated social and structural determinants of poor health. Future research should also explore the extent to which structural racism should be integrated within a comprehensive approach, as many of these communities have likely long experienced societal and structural inequities, such as historical disinvestment.

There are additional limitations based on our methodological approach and focus on what was implemented and actionable for California’s COVID-19 response, including use of data sources. First, HPI is comprised of place-based measures, rather than individual measures. As mentioned in Maizlish et al. (2019), this means that HPI is subject to the limitations of an ecologic design [29]. Second, at the time of writing, HPI is only available for California and Utah. Agencies that do not have HPI for their jurisdictions can consider other place-based indexes, such as CDC’s SVI, which is available at different geographic levels across the US [47]. It is worthwhile to note that HPI and its constituents have been shown to be typically highly but not perfectly correlated with alternative measures recommended federally for examining vulnerability, such as the CDC’s SVI [29, 43, 61]. However, because SVI incorporates race and ethnicity variables, it cannot be utilized for California state policy.

Third, our analysis was conducted at the census tract and ZIP Code levels, which provides more granularity compared to analyses at the county or country level. However, some census tracts located in higher population density or more diverse areas may benefit from analysis conducted at a higher level of data resolution, such as the census block. For example, quantitative data at the census tract level can sometimes obscure variation from small pockets of struggling neighborhoods next to more affluent ones.

Fourth, we elected to present crude rates and rate ratios, without adjusting them for age, race/ethnicity, or other variables. There are two main and interlinked reasons for this. First, although adjusting the outcomes by age and/or race/ethnicity, for example, would help draw comparisons across HPI quartiles, we did not have individual-level age information, and there were high rates of missingness for race/ethnicity (approximately 25–75%, as shown in **S2 Fig**). This data missingness prevented us from computing age- or race/ethnicity-specific rates in the study population, despite having access to age- or race/ethnicity-specific weights derived from ACS at the census tract level. However, using 2016–2020 ACS 5-year estimates, we examined age group distributions at the census tract level for California statewide and by HPI quartiles (**S5 Table**). Examining the share of total population by age groups and assuming that risk of COVID-19-related mortality is highest for census tracts with higher shares of people 65 years or older (with risk decreasing with age), we would expect that age-adjusting mortality rates would make the disparities in the crude mortality rates across HPI quartiles more pronounced. Anticipating how race/ethnicity-adjusting mortality rates would change the disparities presented is more complex, warranting future research. Further, adjusting our outcomes would have deterred from representing what the state implemented in its pandemic policy. Given how California’s Proposition 209 prohibits state agencies from using race as a basis for public policy [63], other non-California agencies may wish to incorporate these variables if the data are available for policy-related purposes, monitoring activities, and health-related resource allocation. However, we suggest understanding the question of interest before adjusting outcomes for key variables, since adjusting can obscure important variation, make results harder to interpret, render results more difficult to act upon, and would require epidemiological or statistical capacity.

Evaluating the public health needs using ABSMs can enable comparisons across diseases and geographies, facilitating targeted interventions with the potential to address common SDOH in prioritized communities. It remains to be understood what the extent of the effects of the equity-focused policy on COVID-19 outcomes were at the local levels. Because counties had the discretion to designate county-level funding to different activities in the county investment plans, it would be worth understanding whether this approach was helpful and if it can be utilized or improved upon for future public health responses.

## Conclusions

Socioeconomically disadvantaged communities and racial/ethnic minority groups in the US have experienced longstanding and pervasive health disparities. As these disparities were illuminated during the COVID-19 pandemic, California implemented one of the largest at-scale equity-focused COVID-19 response and reopening policies in the US, which included at its core the use of an ABSM called the California HPI. HPI was a valuable and actionable tool for tracking disparities, guiding policies, and allocating resources in a pandemic response for the most populous US state. California’s experience offers important policy implications for other settings to consider, including how a place-based approach provided an SDOH lens that circumvented challenges inherent in public health practice, including incomplete information when using individual-level data, issues with not accounting for intersecting determinants of health outcomes of interest, and how to operationally target interventions at a large scale. The approach also supported efforts to analyze multiple dimensions of data on health outcomes, define equity metrics essential to monitoring disparities in the pandemic response, and facilitate the targeting of public health interventions and policies. Additional research is needed to evaluate the implementation of these policies on equity and inform scale-up of similar approaches for a broader array of public health priorities.

## Supporting information

S1 Fig Map of statewide HPI 2.0 score percentile ranking of California census tracts. (PDF)

S2 Fig Using HPI at census tract level provided useful information for prioritizing populations: Weekly cases, hospitalizations, and deaths by HPI quartiles and race groups from case surveillance. (PDF)

S3 Fig Average population percentage comprised of different race/ethnicity groups by HPI percentile among census tracts. (PDF)

S1 Table HPI version 2.0 constituents. (PDF)

S2 Table Summary statistics for tracts in California, large counties, and small counties by Health Equity Quartile (HEQ; within-county quartile 1) or non-HEQ (within-county quartiles 2–4). (PDF)

S3 Table Summary of statewide HPI quartiles and COVID-19 outcomes by California county and county size. (PDF)

S4 Table Unadjusted monthly test, case, and death rate ratio (RR) results. (PDF)

S5 Table Total population and percent of total population for age groups in California: Statewide and across HPI quartiles. (PDF)

S1 Text Detailed background on the Blueprint for a Safer Economy. (PDF)
