# Supplementary material for: The integration of health equity into policy to reduce disparities: Lessons from California during the COVID-19 pandemic
Source: PLoS One. 2025 Mar 6;20(3):e0316517. doi: 10.1371/journal.pone.0316517 (PMC11884665; doi:10.1371/journal.pone.0316517)
Supplement: S1 Fig — (PDF) [file pone.0316517.s001.pdf]

S2 Figure. Map of statewide HPI 2.0 score percentile ranking of California census tracts.

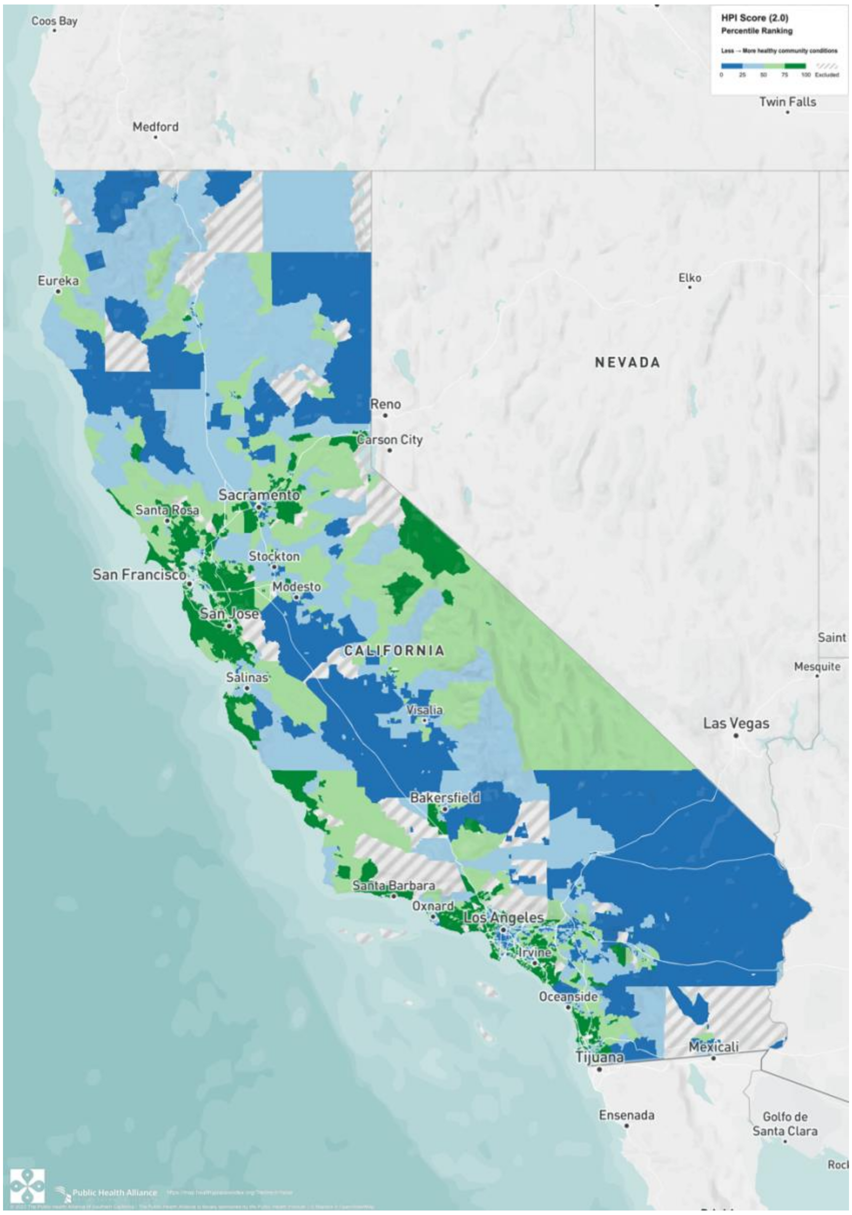

Note: Reprinted from the California Healthy Places Index (HPI) website (https://map.healthyplacesindex.org) under a CC BY license, with permission from the Public Health Alliance of Southern California (data) and Axis Maps (visualization), original copyright 2022.
