# Supplementary material for: The integration of health equity into policy to reduce disparities: Lessons from California during the COVID-19 pandemic
Source: PLoS One. 2025 Mar 6;20(3):e0316517. doi: 10.1371/journal.pone.0316517 (PMC11884665; doi:10.1371/journal.pone.0316517)
Supplement: S2 Fig — (PDF) [file pone.0316517.s002.pdf]

S6 Figure. Using HPI at census tract level provided useful information for prioritizing populations: Weekly cases, hospitalizations, and deaths by HPI quartiles and race groups from case surveillance

a. Frequency and Proportion of Weekly COVID-19 Cases by HPI Quartiles

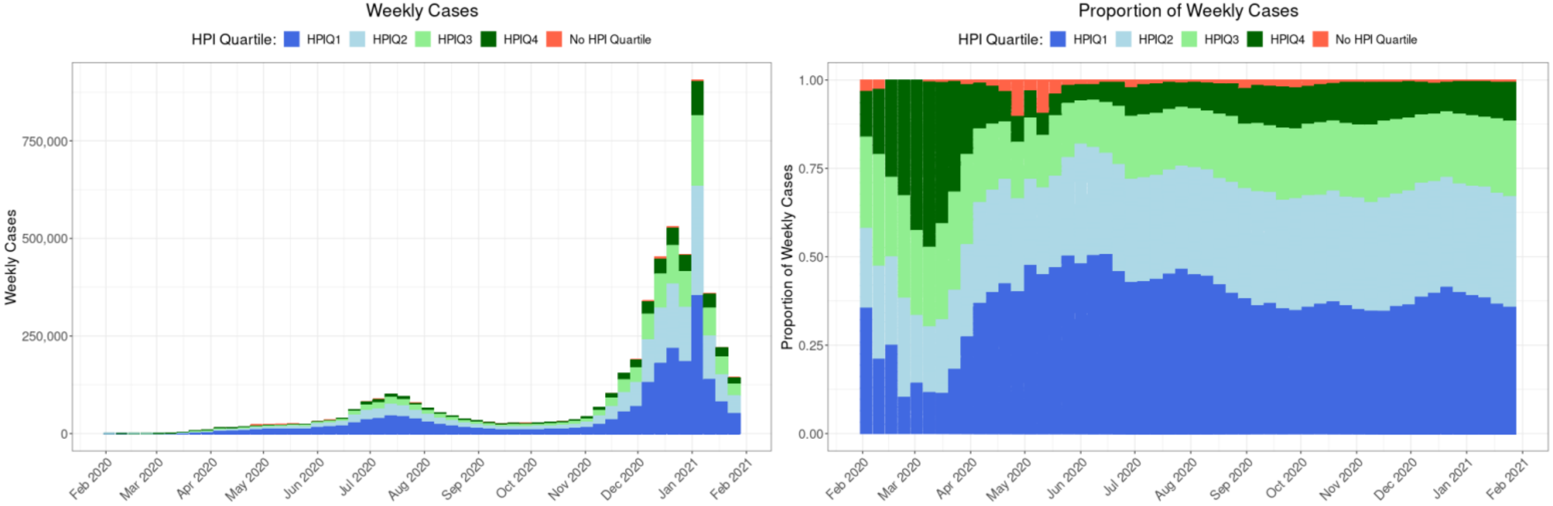

b. Frequency and Proportion of Weekly COVID-19 Cases by Race

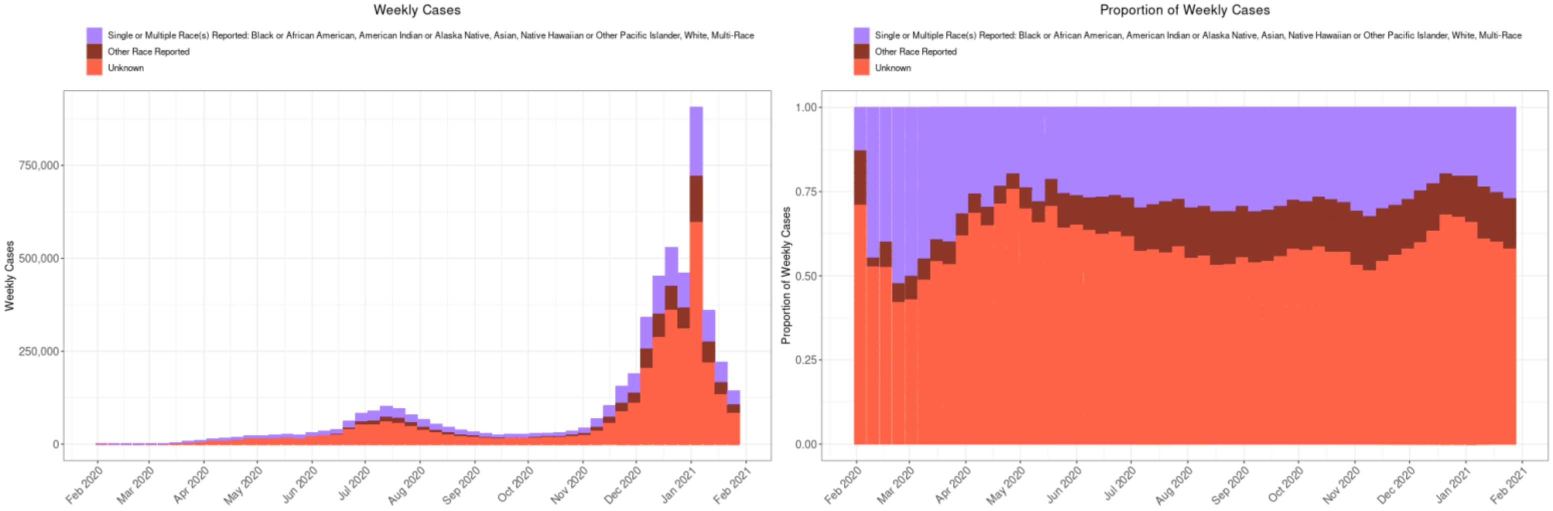

Note: HPI is California Healthy Places index (version 2.0). Race groups are self-reported at point-of-testing and derived from CDPH surveillance data. Ethnicity is not included in race groups listed. Race group of “other” refers to those who do not fall under any listed race group. Race group of “unknown” includes those who declined to state or whose race information is missing.

Version: 20 November 2024

c. Frequency and Proportion of Weekly Hospitalizations with COVID-19 by HPI Quartiles

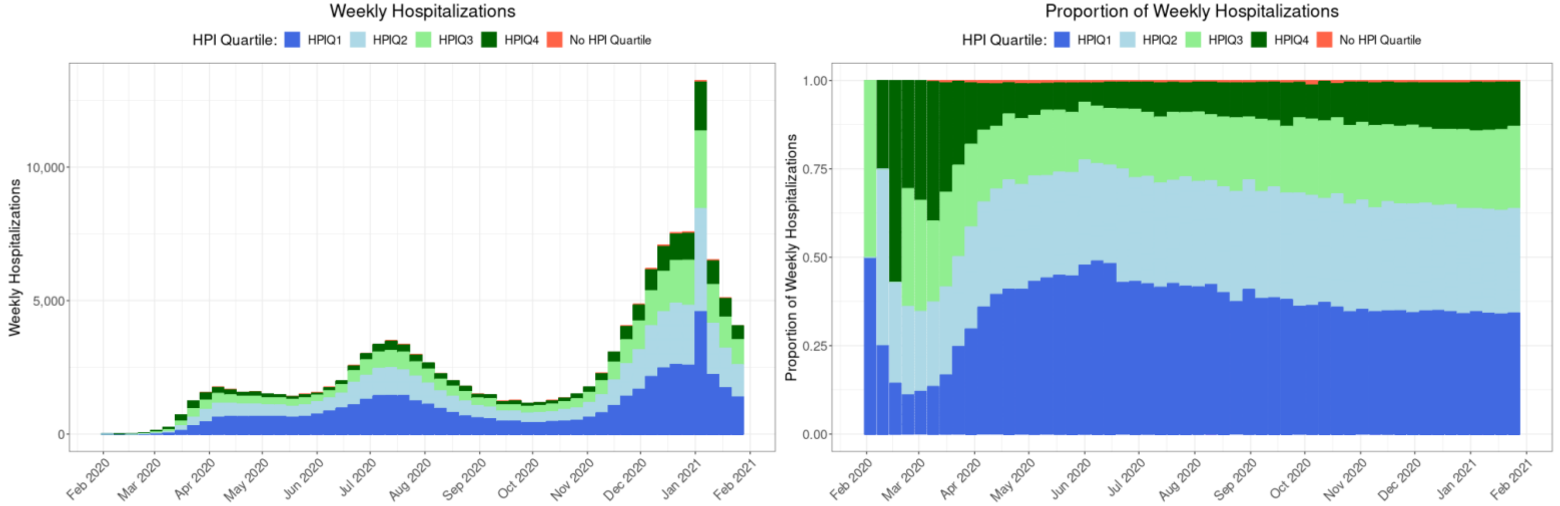

d. Frequency and Proportion of Weekly Hospitalizations with COVID-19 by Race

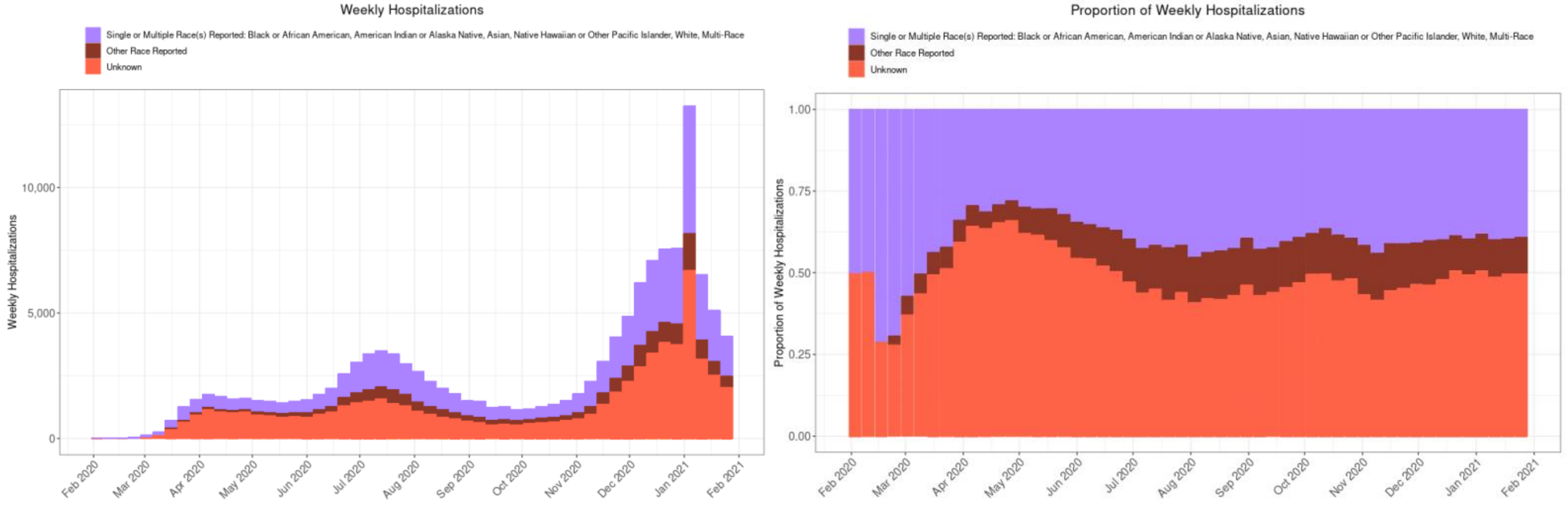

Note: HPI is California Healthy Places index (version 2.0). Race groups are self-reported at point-of-testing and derived from CDPH surveillance data. Ethnicity is not included in race groups listed. Race group of “other” refers to those who do not fall under any listed race group. Race group of “unknown” includes those who declined to state or whose race information is missing.

e. Frequency and Proportion of Weekly Deaths with COVID-19 by HPI Quartiles

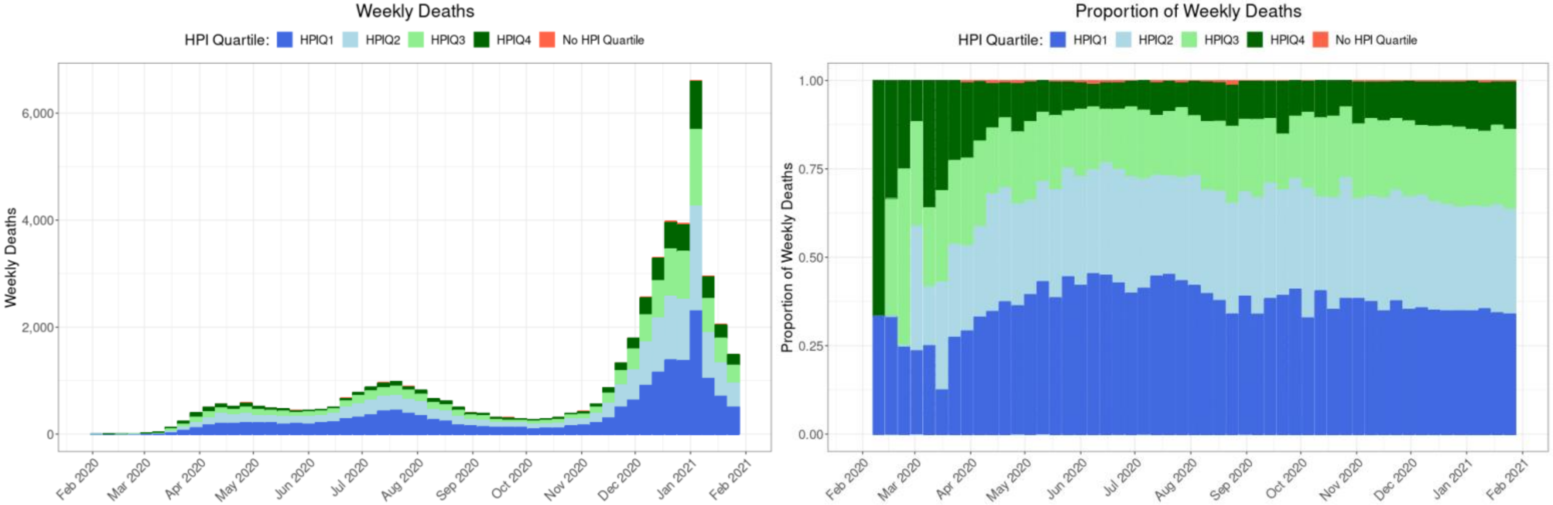

f. Frequency and Proportion of Weekly Deaths with COVID-19 by Race

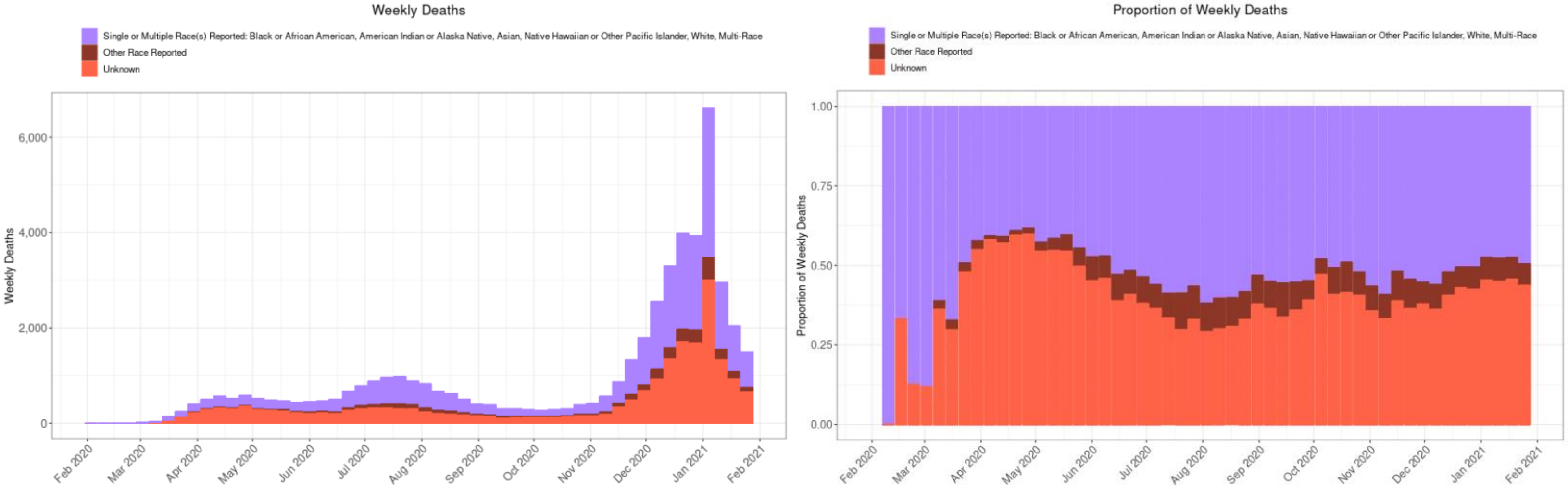

Note: HPI is California Healthy Places index (version 2.0). Race groups are self-reported at point-of-testing and derived from CDPH surveillance data. Ethnicity is not included in race groups listed. Race group of “other” refers to those who do not fall under any listed race group. Race group of “unknown” includes those who declined to state or whose race information is missing.
