# Supplementary material for: The integration of health equity into policy to reduce disparities: Lessons from California during the COVID-19 pandemic
Source: PLoS One. 2025 Mar 6;20(3):e0316517. doi: 10.1371/journal.pone.0316517 (PMC11884665; doi:10.1371/journal.pone.0316517)
Supplement: S3 Fig — (PDF) [file pone.0316517.s003.pdf]

S7 Figure. Average population percentage comprised of different race/ethnicity groups by HPI percentile among census tracts.

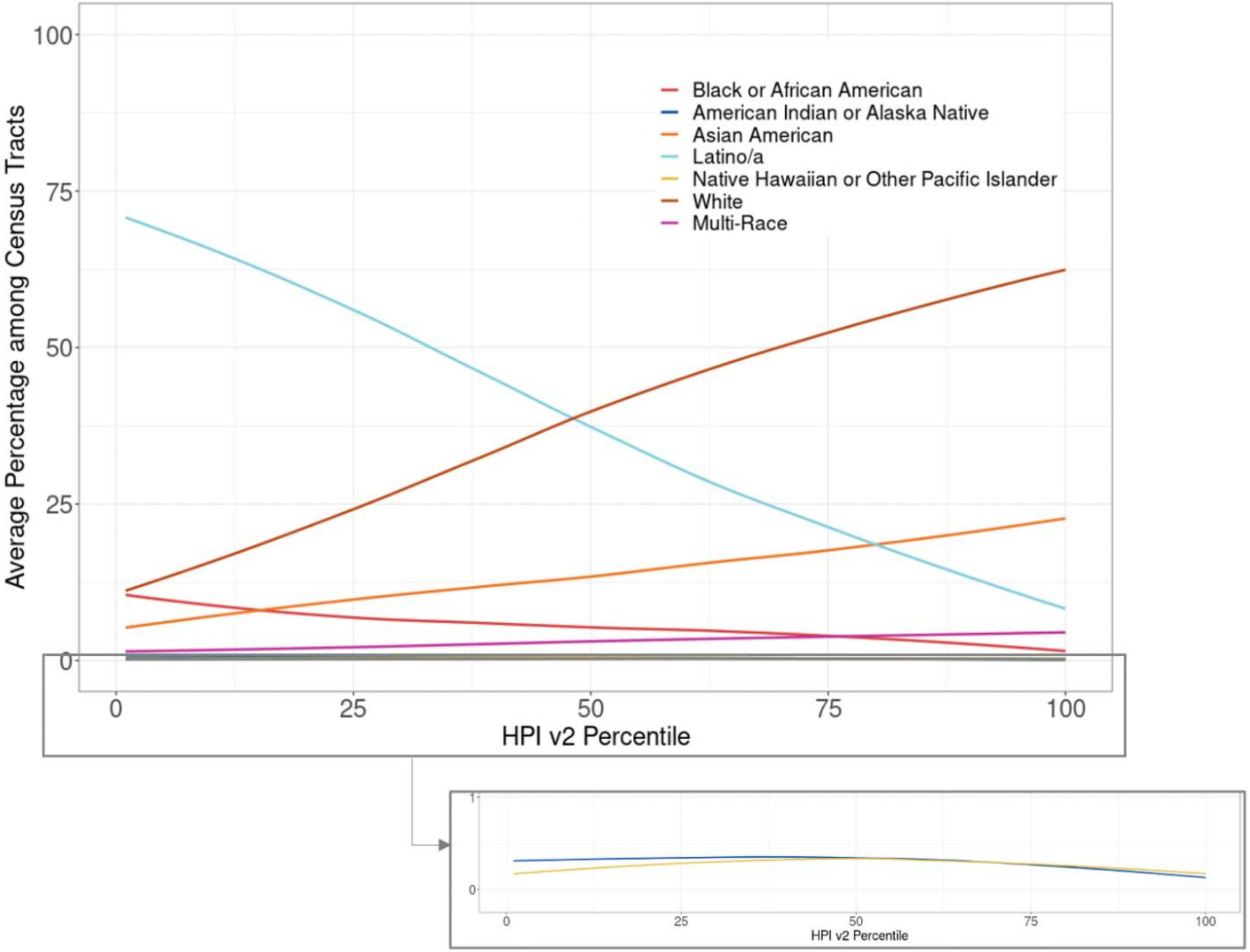

Note: Percentage of population by race/ethnicity groups at census tract level are 2019 ACS 5-year population estimates. The bottom box expands the visual field for the average population percentage comprised of two groups: (1) American Indian or Alaska Native, and (2) Native Hawaiian or Other Pacific Islander.

Version: 20 November 2024
