# Supplementary material for: The integration of health equity into policy to reduce disparities: Lessons from California during the COVID-19 pandemic
Source: PLoS One. 2025 Mar 6;20(3):e0316517. doi: 10.1371/journal.pone.0316517 (PMC11884665; doi:10.1371/journal.pone.0316517)
Supplement: S1 Table — (PDF) [file pone.0316517.s004.pdf]

S1 Table. HPI version 2.0 Constituents.

Policy Action Areas (Domains), Indicators, and their Data Sources.

	**Indicator Policy Domains**<br><br>Indicator	**Data Source† , Year HPI 2.0**
**1**	**Economic**	
	Percent of the population with an income exceeding 200% of federal poverty level	ACS, 2011-2015
	Percentage of population aged 25-64 who are employed	ACS, 2011-2015
	Median Household Income	ACS, 2011-2015
**2**	**Education**	
	Percentage of population over age 25 with a bachelor's education or higher	ACS, 2011-2015
	Percentage of 15-17-year-olds enrolled in school	ACS, 2011-2015
	Percentage of 3- and 4-year-olds enrolled in pre-school	ACS, 2011-2015
**3**	**Social**	
	Percentage of registered voters voting in the 2020 general election	UC Berkeley, 2012
	Percentage of family households with children under 18 with two parents	ACS, 2011-2015
**4**	**Transportation**	
	Percentage of households with access to an automobile	ACS, 2011-2015
	Percentage of workers (16 years and older) commuting by walking, cycling, or transit (excluding working from home)	ACS, 2011-2015
**5**	**Healthcare Access**	
	Percentage of adults aged 18 to 64 years currently insured	ACS, 2011-2015
**6**	**Neighborhood**	
	Percentage of the population living within ½ -mile of a park, beach, or open space greater than 1 acre	GreenInfo, 2012
	Population-weighted percentage of the census tract area with tree canopy	NLCD, 2011
	Percentage of the population residing within ¼ mile of an off-site sales alcohol outlet	ABC, 2014
	Percentage of the urban and small-town population residing less than 1/2 mile from a supermarket/large grocery store, and the percent of the rural population living less than 1 miles from a supermarket/large grocery store	USDA, 2015
	Combined employment density for retail, entertainment, supermarkets, and educational uses (jobs/acre)	USEPA, 2006-2010
**7**	**Housing**	
	Percentage of occupied housing units occupied by property owners	ACS, 2011-2015
	Percent of households with complete kitchen facilities and plumbing	CHAS, 2010-2014
	Percentage of low-income homeowners paying more than 50% of income on housing	CHAS, 2010-2014
	Percentage of low-income renter households paying more than 50% of income on housing	CHAS, 2010-2014
	Percentage of households with less or equal to 1 occupant per room	ACS, 2011-2015
**8**	**Clean Environment**	
	Annual average spatial distribution of gridded diesel PM emissions from on-road and non-road sources 2012 (tons/year).	CalEPA, 2012
	CalEnviroScreen 3.0 drinking water contaminant index for selected contaminants	CalEPA, 2005-2013
	Mean of summer months (May-October) of the daily maximum 8-hour ozone concentration (ppm), averaged over three years (2012 to 2014)	CalEPA, 2012-2014
	Annual mean concentration of PM2.5 (μg/m3) over three years (2012 to 2014).	CalEPA, 2012-2014

Note: Indicators are for the California Healthy Places Index (HPI) version 2.0, developed by the Public Health Alliance of Southern California. ABC is Alcoholic Beverage Commission. ACS is American Community Survey. CHAS is Comprehensive Housing Assessment System. CalEPA is California Environmental Protection Agency. GreenInfo is CaLANDS. NLCD is National Land Cover Database. USDA FARA is U.S. Department of Agriculture Food Access Research Atlas. USEPA is U.S. Environmental Protection Agency. LODES is LEHD Origin-Destination Employment Statistics. UC Berkeley is University of California, Berkeley.
