# Supplementary material for: The integration of health equity into policy to reduce disparities: Lessons from California during the COVID-19 pandemic
Source: PLoS One. 2025 Mar 6;20(3):e0316517. doi: 10.1371/journal.pone.0316517 (PMC11884665; doi:10.1371/journal.pone.0316517)
Supplement: S2 Table — (PDF) [file pone.0316517.s005.pdf]

S4 Table. Summary statistics for tracts in California, large counties, and small counties by Health Equity Quartile (HEQ; within-county quartile 1) or non-HEQ (within-county quartiles 2-4)

	**Tracts in California**				**Tracts in Large Counties**				**Tracts in Small Counties**			
	Non-HEQ		HEQ		Non-HEQ		HEQ		Non-HEQ		HEQ	
	N	*Mean*	*N*	*Mean*	*N*	*Mean*	*N*	*Mean*	*N*	*Mean*	*N*	*Mean*
Population (2019)	5,824	5,008.87	1,969	4,884.81	5,679	5,021.08	1,910	4,905.98	145	4,530.59	59	4,199.31
Life expectancy at birth	5,801	81.02	1,966	78.05	5,661	81.07	1,907	78.08	140	79.23	59	77.34
Proportion 65 years or older	5,824	0.14	1,969	0.10	5,679	0.14	1,910	0.10	145	0.19	59	0.18
Proportion White	5,824	0.45	1,969	0.20	5,679	0.44	1,910	0.18	145	0.70	59	0.63
Proportion Black	5,824	0.05	1,969	0.08	5,679	0.05	1,910	0.08	145	0.01	59	0.02
Proportion Asian	5,824	0.15	1,969	0.10	5,679	0.15	1,910	0.11	145	0.03	59	0.03
Proportion Native American	5,824	0.00	1,969	0.00	5,679	0.00	1,910	0.00	145	0.02	59	0.03
Proportion Pacific Islander	5,824	0.00	1,969	0.00	5,679	0.00	1,910	0.00	145	0.00	59	0.00
Proportion Other race	5,824	0.00	1,969	0.00	5,679	0.00	1,910	0.00	145	0.00	59	0.00
Proportion Multiple races	5,824	0.03	1,969	0.02	5,679	0.03	1,910	0.02	145	0.03	59	0.04
Proportion Latino/a	5,824	0.32	1,969	0.58	5,679	0.32	1,910	0.60	145	0.20	59	0.25
**<u>Economic</u>**												
Proportion with an income exceeding 200% of federal poverty level	5,801	0.75	1,966	0.50	5,661	0.76	1,907	0.49	140	0.68	59	0.56
Proportion aged 25-64 who are employed	5,801	0.74	1,966	0.68	5,661	0.74	1,907	0.68	140	0.66	59	0.60
Per capita income	5,801	43,629.67	1,966	21,610.49	5,661	43,918.08	1,907	21,472.57	140	31,967.77	59	26,068.36
**<u>Education</u>**												
Proportion over age 25 with a bachelor's education or higher	5,801	0.39	1,966	0.16	5,661	0.39	1,907	0.16	140	0.23	59	0.17
Proportion of 15-17-year-olds enrolled in school	5,801	0.98	1,966	0.97	5,661	0.98	1,907	0.97	140	0.97	59	0.96
Proportion of 3- and 4-year-olds enrolled in pre-school	5,801	0.56	1,966	0.43	5,661	0.56	1,907	0.43	140	0.50	59	0.45
**<u>Social</u>**												
Proportion of households who completed census forms (2020)	5,801	0.73	1,966	0.63	5,661	0.73	1,907	0.63	140	0.60	59	0.55
Proportion of registered voters voting in the 2020 general election	5,801	0.81	1,966	0.68	5,661	0.81	1,907	0.68	140	0.81	59	0.77
**<u>Transportation</u>**												
Proportion of households with access to an automobile	5,801	0.95	1,966	0.88	5,661	0.95	1,907	0.88	140	0.96	59	0.92
Proportion of workers (16 years and older) commuting by walking, cycling, or transit (excluding working from home)	5,801	0.08	1,966	0.12	5,661	0.08	1,907	0.12	140	0.04	59	0.07

(continued on next page)

S4 Table continued.

	Tracts in California				Tracts in Large Counties				Tracts in Small Counties			
	Non-HEQ		HEQ		Non-HEQ		HEQ		Non-HEQ		HEQ	
	N	*Mean*	*N*	*Mean*	*N*	*Mean*	*N*	*Mean*	*N*	*Mean*	*N*	*Mean*
**Healthcare Access**												
Proportion of adults aged 18 to 64 years currently insured	5,801	0.92	1,966	0.82	5,661	0.92	1,907	0.82	140	0.90	59	0.88
**Neighborhood**												
Proportion living within ½ -mile of a park, beach, or open space >1 acre	5,801	0.77	1,966	0.76	5,661	0.78	1,907	0.77	140	0.48	59	0.61
Population-weighted percentage of the census tract area with tree canopy	5,801	0.09	1,966	0.06	5,661	0.08	1,907	0.06	140	0.24	59	0.22
Employment density (jobs/acre)	5,801	6.69	1,966	7.79	5,661	6.82	1,907	7.98	140	1.09	59	1.74
**Housing**												
Proportion of occupied housing units occupied by property owners	5,801	0.61	1,966	0.37	5,661	0.61	1,907	0.36	140	0.69	59	0.58
Proportion of households with complete kitchen facilities and plumbing	5,801	0.99	1,966	0.98	5,661	0.99	1,907	0.98	140	0.99	59	0.98
Proportion of low-income homeowners paying >50% income on housing	5,801	0.11	1,966	0.16	5,661	0.11	1,907	0.16	140	0.10	59	0.11
Proportion of low-income renter households paying >50% income on housing	5,801	0.23	1,966	0.32	5,661	0.23	1,907	0.32	140	0.22	59	0.27
Proportion of households with less or equal to 1 occupant per room	5,801	0.94	1,966	0.82	5,661	0.94	1,907	0.82	140	0.96	59	0.95
**Clean Environment**												
Annual average spatial distribution of gridded diesel PM emissions from on-road and non-road sources 2016 (tons/year)	5,801	0.19	1,966	0.31	5,661	0.20	1,907	0.32	140	0.04	59	0.07
Drinking water contaminant index for selected contaminants (CalEnviroScreen 4.0)	5,801	475.91	1,966	481.64	5,661	477.39	1,907	483.21	140	415.91	59	430.99
Mean of summer months (May-October) of the daily max 8-hour ozone concentration (ppm), averaged over 2017 to 2019	5,801	0.05	1,966	0.05	5,661	0.05	1,907	0.05	140	0.05	59	0.05
Annual mean concentration of PM2.5 (μg/m3) over three years (2015-17)	5,801	10.13	1,966	10.32	5,661	10.22	1,907	10.44	140	6.52	59	6.50
