# Supplementary material for: The integration of health equity into policy to reduce disparities: Lessons from California during the COVID-19 pandemic
Source: PLoS One. 2025 Mar 6;20(3):e0316517. doi: 10.1371/journal.pone.0316517 (PMC11884665; doi:10.1371/journal.pone.0316517)
Supplement: S3 Table — (PDF) [file pone.0316517.s006.pdf]

S5 Table. Summary of statewide HPI quartiles and COVID-19 outcomes by California county and county size.

County	Number of CTs	Population (2019 ACS 5-year)	Number of CTs w/o HPI score	Row %	Number of CTs with HPI score	Row %	Number of CTs in HPIq1	Row %	Number of CTs in HPIq2	Row %	Number of CTs in HPIq3	Row %	Number of CTs in HPIq4	Row %	Tests conducted	Positives	Cases reported	Deaths reported	Tests per 100k county pop.	Test Positivity Rate (%)	Cases per 100k county pop.	Deaths per 100k county pop.
**All Counties**	8,057	39,283,497	264	3.3	7,793	96.7	1,949	24.2	1,948	24.2	1,948	24.2	1,948	24.2	37,344,862	3,598,586	3,127,040	54,266	96,274.9	9.6	8,061.5	139.9
**Large Counties**	**7,839**	**38,352,825**	**250**	**3.2**	**7,589**	**96.8**	**1,905**	**24.3**	**1,863**	**23.8**	**1,885**	**24.0**	**1,936**	**24.7**	**36,765,826**	**3,552,830**	**3,083,629**	**53,627**				
Alameda	361	1,656,754	9	2.5	352	97.5	28	7.8	66	18.3	91	25.2	167	46.3	1,491,216	81,141	71,692	1,257	91,059.4	5.4	4,377.8	76.8
Butte	51	225,817	0	0.0	51	100.0	14	27.5	19	37.3	16	31.4	2	3.9	136,281	9,938	9,798	194	60,350.2	7.3	4,338.9	85.9
Contra Costa	208	1,142,251	4	1.9	204	98.1	16	7.7	30	14.4	51	24.5	107	51.4	979,134	60,897	56,904	774	86,007.8	6.2	4,998.5	68.0
El Dorado	43	188,563	3	7.0	40	93.0	0	0.0	12	27.9	15	34.9	13	30.2	103,434	7,242	7,468	109	55,141.3	7.0	3,981.2	58.1
Fresno	199	984,521	7	3.5	192	96.5	108	54.3	35	17.6	40	20.1	9	4.5	707,003	93,714	82,116	1,579	72,866.8	13.3	8,463.2	162.7
Humboldt	31	135,940	2	6.5	29	93.5	4	12.9	11	35.5	14	45.2	0	0.0	79,009	2,519	2,447	30	58,690.4	3.2	1,817.7	22.3
Imperial	31	180,701	5	16.1	26	83.9	16	51.6	5	16.1	3	9.7	2	6.5	144,082	24,447	22,533	690	85,410.8	17.0	13,357.4	409.0
Kern	151	887,641	10	6.6	141	93.4	75	49.7	34	22.5	23	15.2	9	6.0	571,342	86,516	77,414	1,115	66,622.1	15.1	9,027.0	130.0
Kings	27	150,691	2	7.4	25	92.6	15	55.6	7	25.9	3	11.1	0	0.0	143,280	14,930	12,960	192	103,314.0	10.4	9,345.0	138.4
Los Angeles	2,346	10,081,570	85	3.6	2,261	96.4	770	32.8	613	26.1	487	20.8	391	16.7	13,213,737	1,359,076	1,093,882	20,757	132,648.2	10.3	10,981.1	208.4
Madera	23	155,433	2	8.7	21	91.3	9	39.1	8	34.8	4	17.4	0	0.0	86,072	12,175	11,419	164	59,182.0	14.1	7,851.6	112.8
Marin	56	259,943	3	5.4	53	94.6	0	0.0	2	3.6	7	12.5	44	78.6	287,496	10,619	9,811	204	112,276.8	3.7	3,831.5	79.7
Merced	49	271,382	0	0.0	49	100.0	36	73.5	11	22.4	2	4.1	0	0.0	222,004	25,277	24,357	362	81,805.0	11.4	8,975.2	133.4
Monterey	94	433,410	6	6.4	88	93.6	18	19.1	29	30.9	26	27.7	15	16.0	333,805	36,853	33,310	447	79,716.7	11.0	7,954.8	106.7
Napa	40	139,623	3	7.5	37	92.5	0	0.0	6	15.0	19	47.5	12	30.0	167,072	8,479	8,103	73	122,204.6	5.1	5,926.9	53.4
Orange	583	3,168,044	11	1.9	572	98.1	58	9.9	141	24.2	156	26.8	217	37.2	2,196,789	260,200	220,155	4,473	69,603.6	11.8	6,975.4	141.7
Placer	85	385,512	6	7.1	79	92.9	2	2.4	11	12.9	31	36.5	35	41.2	230,101	18,289	17,948	269	60,247.9	7.9	4,699.4	70.4
Riverside	453	2,411,439	11	2.4	442	97.6	160	35.3	153	33.8	105	23.2	24	5.3	1,931,059	291,693	252,762	3,715	80,995.3	15.1	10,601.7	155.8
Sacramento	317	1,524,553	10	3.2	307	96.8	65	20.5	93	29.3	92	29.0	57	18.0	1,061,886	90,101	82,548	1,633	70,474.8	8.5	5,478.5	108.4
San Bernardino	369	2,149,031	10	2.7	359	97.3	163	44.2	105	28.5	66	17.9	25	6.8	1,897,348	295,600	253,715	4,670	89,931.3	15.6	12,025.7	221.4
San Diego	628	3,316,073	12	1.9	616	98.1	107	17.0	154	24.5	183	29.1	172	27.4	2,415,924	201,819	228,341	2,891	73,478.6	8.4	6,944.8	87.9
San Francisco	197	874,961	8	4.1	189	95.9	18	9.1	13	6.6	46	23.4	112	56.9	1,215,439	38,661	31,073	462	139,933.2	3.2	3,577.4	53.2
San Joaquin	139	742,603	1	0.7	138	99.3	53	38.1	48	34.5	31	22.3	6	4.3	527,440	65,247	58,178	1,343	71,423.0	12.4	7,878.1	181.9
San Luis Obispo	54	282,165	6	11.1	48	88.9	1	1.9	8	14.8	27	50.0	12	22.2	244,507	14,635	14,053	217	92,441.2	6.0	5,313.0	82.0
San Mateo	158	767,423	2	1.3	156	98.7	0	0.0	13	8.2	22	13.9	121	76.6	907,905	43,705	35,783	476	118,305.7	4.8	4,662.7	62.0
Santa Barbara	90	444,829	7	7.8	83	92.2	15	16.7	9	10.0	26	28.9	33	36.7	362,368	29,675	26,801	391	84,651.0	8.2	6,260.8	91.3
Santa Clara	372	1,927,470	7	1.9	365	98.1	12	3.2	54	14.5	87	23.4	212	57.0	2,196,056	108,938	98,712	1,534	114,717.2	5.0	5,156.5	80.1
Santa Cruz	53	273,962	2	3.8	51	96.2	3	5.7	9	17.0	20	37.7	19	35.8	238,360	13,763	13,704	197	90,467.4	5.8	5,201.2	74.8
Shasta	48	179,212	2	4.2	46	95.8	8	16.7	21	43.8	15	31.3	2	4.2	110,865	6,898	9,139	171	62,968.2	6.2	5,190.7	97.1
Solano	96	441,829	3	3.1	93	96.9	20	20.8	23	24.0	31	32.3	19	19.8	352,971	25,692	25,229	244	81,297.7	7.3	5,810.8	56.2
Sonoma	100	499,772	1	1.0	99	99.0	0	0.0	21	21.0	51	51.0	27	27.0	392,990	26,332	24,557	368	78,633.9	6.7	4,913.6	73.6
Stanislaus	94	543,194	0	0.0	94	100.0	43	45.7	35	37.2	16	17.0	0	0.0	393,875	50,065	45,408	818	72,510.9	12.7	8,359.4	150.6
Tulare	78	461,898	2	2.6	76	97.4	48	61.5	19	24.4	9	11.5	0	0.0	358,306	45,358	41,269	768	77,880.6	12.7	8,970.1	166.9
Ventura	174	847,263	7	4.0	167	96.0	17	9.8	34	19.5	56	32.2	60	34.5	896,754	81,215	68,771	835	106,447.2	9.1	8,163.3	99.1
Yolo	41	217,352	1	2.4	40	97.6	3	7.3	11	26.8	14	34.1	12	29.3	169,916	11,121	11,269	205	81,398.4	6.5	5,398.4	98.2
**Small Counties**	**218**	**930,672**	**14**	**6.4**	**204**	**93.6**	**44**	**20.2**	**85**	**39.0**	**63**	**28.9**	**12**	**5.5**	**579,036**	**45,756**	**43,411**	**639**				
70,001 - 106,000																						
Mendocino	21	87,224	1	4.8	20	95.2	4	19.0	9	42.9	6	28.6	1	4.8	55,391	2,885	2,828	32	63,504.3	5.2	3,242.2	36.7
Nevada	20	99,244	0	0.0	20	100.0	0	0.0	5	25.0	10	50.0	5	25.0	63,474	3,083	3,290	79	63,957.5	4.9	3,315.1	79.6
Sutter	21	96,109	0	0.0	21	100.0	6	28.6	7	33.3	8	38.1	0	0.0	70,611	8,921	8,056	111	73,469.7	12.6	8,382.1	115.5
Yuba	14	76,360	0	0.0	14	100.0	8	57.1	4	28.6	2	14.3	0	0.0	44,249	5,342	4,788	48	57,947.9	12.1	6,270.3	62.9
35,001 - 70,000																						
Amador	9	38,429	1	11.1	8	88.9	0	0.0	2	22.2	6	66.7	0	0.0	24,587	1,335	1,205	44	73,781.7	5.4	3,616.0	132.0
Calaveras	10	45,514	1	10.0	9	90.0	2	20.0	4	40.0	3	30.0	0	0.0	18,760	1,305	1,214	24	41,636.1	7.0	2,694.4	53.3
Lake	15	64,195	0	0.0	15	100.0	8	53.3	6	40.0	1	6.7	0	0.0	37,586	2,785	2,486	54	58,549.7	7.4	3,872.6	84.1
San Benito	11	60,376	0	0.0	11	100.0	0	0.0	4	36.4	5	45.5	2	18.2	53,548	5,563	4,981	49	88,690.9	10.4	8,250.0	81.2
Siskiyou	14	43,468	3	21.4	11	78.6	3	21.4	7	50.0	1	7.1	0	0.0	17,051	933	912	8	42,127.2	5.5	2,253.2	19.8
Tehama	11	63,912	0	0.0	11	100.0	2	18.2	8	72.7	1	9.1	0	0.0	37,890	3,768	3,986	57	59,284.6	9.9	6,236.7	89.2
Tuolumne	11	54,045	1	9.1	10	90.9	0	0.0	4	36.4	6	54.5	0	0.0	39,403	2,270	1,952	43	76,457.2	5.8	3,787.6	83.4
≤35,000																						
Alpine	1	1,039	1	100.0	0	0.0	0	0.0	0	0.0	0	0.0	0	0.0								
Colusa	5	21,454	0	0.0	5	100.0	0	0.0	5	100.0	0	0.0	0	0.0	8,811	1,224	1,354	10	41,069.3	13.9	6,311.2	46.6
Del Norte	8	27,495	2	25.0	6	75.0	3	37.5	2	25.0	1	12.5	0	0.0	24,386	635	600	3	92,920.3	2.6	2,286.2	11.4
Glenn	6	27,976	0	0.0	6	100.0	2	33.3	4	66.7	0	0.0	0	0.0	16,194	1,858	1,865	23	57,885.3	11.5	6,666.4	82.2
Inyo	6	17,977	0	0.0	6	100.0	0	0.0	1	16.7	4	66.7	1	16.7	10,753	766	907	30	59,815.3	7.1	5,045.3	166.9
Lassen	9	30,818	1	11.1	8	88.9	3	33.3	2	22.2	3	33.3	0	0.0	26,287	1,555	1,333	11	122,049.4	5.9	6,189.1	51.1
Mariposa	6	17,420	0	0.0	6	100.0	0	0.0	2	33.3	3	50.0	1	16.7	11,293	298	280	4	64,827.8	2.6	1,607.3	23.0
Modoc	4	8,907	1	25.0	3	75.0	0	0.0	3	75.0	0	0.0	0	0.0	1,807	88	136	1	23,776.3	4.9	1,789.5	13.2
Mono	3	14,310	0	0.0	3	100.0	0	0.0	0	0.0	1	33.3	2	66.7	7,186	683	643	3	50,216.6	9.5	4,493.4	21.0
Plumas	7	18,660	1	14.3	6	85.7	1	14.3	4	57.1	1	14.3	0	0.0	7,024	361	416	5	40,095.9	5.1	2,374.7	28.5
Sierra	1	3,040	0	0.0	1	100.0	1	100.0	0	0.0	0	0.0	0	0.0	671	19	22	0	22,072.4	2.8	723.7	0.0
Trinity	5	12,700	1	20.0	4	80.0	1	20.0	2	40.0	1	20.0	0	0.0	2,074	79	157	0	17,567.3	3.8	1,329.8	0.0

Note: CT is census tract. HPI is California Healthy Places Index version 2.0. HPIq1, HPIq2, HPIq3, and HPIq4 refer to HPI quartiles 1, 2, 3, and 4, respectively. Population is 2019 ACS 5-year population estimates. Large counties are counties with population greater than 106,000.
