# Supplementary material for: The integration of health equity into policy to reduce disparities: Lessons from California during the COVID-19 pandemic
Source: PLoS One. 2025 Mar 6;20(3):e0316517. doi: 10.1371/journal.pone.0316517 (PMC11884665; doi:10.1371/journal.pone.0316517)
Supplement: S4 Table — (PDF) [file pone.0316517.s007.pdf]

S8 Table. Unadjusted monthly test, case, and death rate ratio (RR) results

Month	HPI Quartile		Monthly Tests RR	Monthly Tests 95% CI	Monthly Cases RR	Monthly Cases 95% CI	Monthly Deaths RR	Monthly Deaths 95% CI
Feb 2020	**Q4**	**(Ref)**	**1.00**		**1.00**		**1.00**	
	Q1		0.95	(0.80, 1.13)	0.45	(0.34, 0.61)	0.00	(0.00, 0.00)
	Q2		0.93	(0.79, 1.10)	0.65	(0.50, 0.84)	0.00	(0.00, 0.00)
	Q3		1.17	(1.00, 1.37)	0.69	(0.54, 0.89)	2.89	(0.30, 27.77)
Mar 2020	**Q4**	**(Ref)**	**1.00**		**1.00**		**1.00**	
	Q1		0.54	(0.54, 0.55)	0.75	(0.72, 0.78)	0.56	(0.40, 0.79)
	Q2		0.68	(0.67, 0.69)	0.81	(0.78, 0.84)	0.72	(0.52, 0.99)
	Q3		0.82	(0.81, 0.83)	0.86	(0.83, 0.90)	0.78	(0.57, 1.06)
Apr 2020	**Q4**	**(Ref)**	**1.00**		**1.00**		**1.00**	
	Q1		1.07	(1.06, 1.08)	4.03	(3.90, 4.16)	2.40	(2.09, 2.75)
	Q2		1.04	(1.04, 1.05)	2.64	(2.55, 2.73)	1.99	(1.73, 2.29)
	Q3		1.00	(0.99, 1.01)	1.65	(1.59, 1.71)	1.53	(1.32, 1.77)
May 2020	**Q4**	**(Ref)**	**1.00**		**1.00**		**1.00**	
	Q1		1.14	(1.13, 1.14)	6.61	(6.41, 6.81)	3.93	(3.42, 4.52)
	Q2		1.08	(1.08, 1.09)	3.98	(3.85, 4.11)	2.41	(2.08, 2.79)
	Q3		1.04	(1.04, 1.05)	2.16	(2.09, 2.24)	1.96	(1.69, 2.28)
Jun 2020	**Q4**	**(Ref)**	**1.00**		**1.00**		**1.00**	
	Q1		1.10	(1.09, 1.10)	4.73	(4.65, 4.82)	5.06	(4.34, 5.91)
	Q2		1.05	(1.05, 1.05)	3.15	(3.10, 3.21)	3.58	(3.06, 4.20)
	Q3		0.99	(0.99, 0.99)	1.90	(1.86, 1.93)	2.11	(1.78, 2.51)
Jul 2020	**Q4**	**(Ref)**	**1.00**	**(1.00, 1.00)**	**1.00**	**(0.98, 1.02)**	**1.00**	**(0.86, 1.16)**
	Q1		1.16	(1.16, 1.17)	4.29	(4.23, 4.35)	5.32	(4.73, 5.99)
	Q2		1.10	(1.10, 1.11)	2.91	(2.87, 2.95)	3.60	(3.19, 4.06)
	Q3		1.02	(1.02, 1.03)	1.83	(1.80, 1.86)	2.14	(1.88, 2.44)
Aug 2020	**Q4**	**(Ref)**	**1.00**		**1.00**	**(0.98, 1.02)**	**1.00**	**(0.87, 1.15)**
	Q1		1.00	(1.00, 1.01)	3.85	(3.78, 3.92)	4.02	(3.60, 4.48)
	Q2		0.99	(0.98, 0.99)	2.80	(2.74, 2.85)	3.03	(2.71, 3.38)
	Q3		0.95	(0.95, 0.96)	1.75	(1.72, 1.79)	2.04	(1.82, 2.30)
Sep 2020	**Q4**	**(Ref)**	**1.00**		**1.00**	**(0.97, 1.03)**	**1.00**	**(0.85, 1.18)**
	Q1		0.85	(0.85, 0.85)	2.73	(2.67, 2.79)	3.25	(2.85, 3.71)
	Q2		0.91	(0.90, 0.91)	2.37	(2.32, 2.43)	2.68	(2.34, 3.07)
	Q3		0.93	(0.93, 0.94)	1.60	(1.56, 1.64)	1.64	(1.41, 1.89)
Oct 2020	**Q4**	**(Ref)**	**1.00**	**(1.00, 1.00)**	**1.00**	**(0.98, 1.02)**	**1.00**	**(0.83, 1.21)**
	Q1		0.79	(0.79, 0.79)	2.81	(2.76, 2.86)	3.04	(2.60, 3.54)
	Q2		0.86	(0.86, 0.87)	2.36	(2.32, 2.41)	2.56	(2.19, 3.00)
	Q3		0.92	(0.91, 0.92)	1.65	(1.62, 1.69)	1.69	(1.43, 2.00)
Nov 2020	**Q4**	**(Ref)**	**1.00**	**(1.00, 1.00)**	**1.00**	**(0.99, 1.01)**	**1.00**	**(0.86, 1.17)**
	Q1		0.83	(0.82, 0.83)	2.59	(2.56, 2.62)	3.26	(2.87, 3.69)
	Q2		0.90	(0.90, 0.90)	2.28	(2.25, 2.30)	2.76	(2.44, 3.14)
	Q3		0.93	(0.93, 0.93)	1.64	(1.62, 1.66)	1.93	(1.69, 2.21)
Dec 2020	**Q4**	**(Ref)**	**1.00**	**(1.00, 1.00)**	**1.00**	**(0.99, 1.01)**	**1.00**	**(0.93, 1.07)**
	Q1		1.13	(1.13, 1.13)	3.16	(3.14, 3.18)	3.03	(2.86, 3.21)
	Q2		1.11	(1.11, 1.11)	2.58	(2.56, 2.60)	2.54	(2.39, 2.70)
	Q3		1.03	(1.02, 1.03)	1.76	(1.75, 1.77)	1.78	(1.68, 1.90)
Jan 2021	**Q4**	**(Ref)**	**1.00**	**(1.00, 1.00)**	**1.00**	**(0.99, 1.01)**	**1.00**	**(0.95, 1.06)**
	Q1		1.13	(1.13, 1.13)	2.79	(2.77, 2.81)	2.77	(2.64, 2.90)
	Q2		1.10	(1.09, 1.10)	2.33	(2.31, 2.35)	2.21	(2.11, 2.32)
	Q3		1.04	(1.03, 1.04)	1.68	(1.67, 1.69)	1.65	(1.57, 1.74)

KEY
<1.00
1.00
>2.00
>3.00
>4.00
>5.00

Note: Unadjusted RR is unadjusted rate ratio. HPI is California Healthy Places Index. Q1, Q2, Q3, and Q4 refer to HPI quartiles 1, 2, 3, and 4, respectively for the state of California.

Version: 20 November 2024
