# Supplementary material for: The integration of health equity into policy to reduce disparities: Lessons from California during the COVID-19 pandemic
Source: PLoS One. 2025 Mar 6;20(3):e0316517. doi: 10.1371/journal.pone.0316517 (PMC11884665; doi:10.1371/journal.pone.0316517)
Supplement: S5 Table — (PDF) [file pone.0316517.s008.pdf]

S9 Table. Total population and percent of total population for age groups in California: Statewide and across HPI quartiles

	California			HPIQ1 (Least Opportunity)			HPIQ2			HPIQ3			HPIQ4 (Most Opportunity)		
	N	*mean*	*sd*	*N*	*Mean*	*SD*	*N*	*mean*	*sd*	*N*	*mean*	*sd*	*N*	*mean*	*sd*
**Total Population**															
0-19 years	8057	1065.6	853.5	1987	1279.8	847.7	1948	1116.3	824.0	1948	970.3	825.4	2174	910.0	865.1
20-49 years	8057	1781.2	1247.0	1987	1859.7	1135.5	1948	1903.4	1163.0	1948	1789.0	1280.0	2174	1592.8	1361.0
50-64 years	8057	790.7	539.2	1987	672.4	405.7	1948	799.3	458.3	1948	842.0	592.5	2174	845.2	637.6
65 years or older	8057	620.3	473.1	1987	457.3	349.4	1948	621.2	434.1	1948	701.8	533.3	2174	695.5	508.6
**Percent of Total Population**															
0-19 years	7247	24.3	7.7	1819	29.3	7.1	1771	24.4	6.6	1728	21.8	6.4	1929	21.8	8.0
20-49 years	7247	41.6	9.6	1819	43.6	6.9	1771	42.7	8.4	1728	41.3	9.9	1929	38.9	11.6
50-64 years	7247	18.8	5.2	1819	16.0	4.2	1771	18.3	4.2	1728	19.8	4.5	1929	21.1	6.1
65 years or older	7247	15.3	8.5	1819	11.1	6.3	1771	14.6	8.1	1728	17.1	9.1	1929	18.2	8.5

Note: HPI is California Healthy Places Index version 2.0. N is the number of census tracts in California. HPIQ{1,2,3,4} are HPI version 2.0 quartiles {1,2,3,4}. Population is based 2016-2020 American Community Survey 5-year estimates.
