# Supplementary material for: The integration of health equity into policy to reduce disparities: Lessons from California during the COVID-19 pandemic
Source: PLoS One. 2025 Mar 6;20(3):e0316517. doi: 10.1371/journal.pone.0316517 (PMC11884665; doi:10.1371/journal.pone.0316517)
Supplement: S1 Text — (PDF) [file pone.0316517.s009.pdf]

S3 Text. Detailed Background on the Blueprint for a Safer Economy

Within the Blueprint, there were three main equity-focused policy efforts, with each leveraging HPI as a structure to guide an equitable COVID-19 reopening and response: (1) the tier reopening framework, (2) the targeted equity investment plans, and (3) the vaccine equity allocation.

<u>First, a tier reopening framework that took effect on August 20, 2020</u> established benchmarks for counties to meet in order to safely "reopen the economy" (e.g., releasing restrictions that would allow for opening up businesses, schools, and other activities). The framework consisted of four tiers, and each week, each county would be assigned a tier that represented their transmission risk (spanning from widespread risk to minimal risk) which then corresponded with the level of reopening restrictions (see **Table A below**).

Within the Blueprint, counties with 106,000 residents or more (designated as "large counties") were assigned a tier each week based on test positivity and adjusted case thresholds. For counties with fewer than 106,000 residents (designated as "small counties"), it was observed that California's case rate metric, which was normalized per 100,000, caused counties with small populations to experience large swings in their daily case rate from a small number of newly reported cases. This would have resulted in a small county moving to more restrictive tiers despite its overall proportion of cases remaining similar to a larger county. Thus, to prevent closing or restricting business sectors and other activities in these counties based on such small numbers of cases, CDPH in collaboration with LHJs decided to use absolute new case numbers (and later on absolute numbers of vaccines) in counties with fewer than 106,000 residents. During the policy, 22 counties had a population less than 100,000, and an additional county which had a population of 106,000 was also included as a small county because it shared a health officer with another small county. Thus, rules for counties with fewer than 106,000 residents adjusted over time, mostly to consider and recognize how a small number of cases could result in large swings to case rates. Ultimately, these "small counties" were assigned a tier each week based on test positivity and case counts by population size (less than 35,000; 35,001 to 70,000; 70,001 to 106,000). In addition to "moving across tiers" when meeting tier-specific thresholds based on county population size, counties also had to meet an equity-focused benchmark.

This equity-focused benchmark was effective from October 6, 2020. Following extended engagement with stakeholders, the equity-focused benchmark was based on the Health Equity Metric (HEM, also the health equity quartile HPI), which was developed using HPI and calculated from the test positivity rates of the lowest quartile of the HPI for each county. Use of the HEM aimed to ensure that the test positivity rate within a county's most disadvantaged census tracts did not substantially trail the county's overall test positivity rate. Specifically, within the tier framework, counties could not move to less restrictive tiers without HEM meeting certain thresholds, but they could also accelerate to a less restrictive tier if the HEM met thresholds for two tiers less restrictive. The equity metric was not considered for moving counties to more restrictive tiers.

Table A. Blueprint for a Safer Economy's Tier Reopening Framework by county size.

a. *Large County Tier Framework (with 106,000 residents or more)*

Higher Risk --> Lower Risk of Community Disease Transmission				
**Measure**	Tier 1 Widespread (Purple)	Tier 2 Substantial (Red)	Tier 3 Moderate (Orange)	Tier 4 Minimal (Yellow)
**Adjusted case rate for tier assignment**	>10	6-10	2-5.9	<2
**Test Positivity**	>8%	5-8%	2-4.9%	<2

Note: Adjusted case rate is the rate of 7-day average cases with 7-day lag per 100,000 population and excludes prison cases. Test positivity is percent of 7-day positive average over tests excluding prison cases.

b. *Small County Tier Framework (with fewer than 106,000)*

**Higher Risk --> Lower Risk of Community Disease Transmission**				
**Case Count for Tier Assignment by County Population Size**	Tier 1 Widespread (Purple)	Tier 2 Substantial (Red)	Tier 3 Moderate (Orange)	Tier 4 Minimal (Yellow)
Fewer than 35,000	≥35	14-34	7-13	<7
35,001-70,000	≥42	21-41	14-20	<14
70,001-106,000	≥49	28-48	21-27	<21
**Test Positivity**	>8%	5-8%	2-4.9%	<2%

Note: Test positivity is percent of 7-day positive average over tests excluding prison cases.

<u>Second, targeted equity investment plans</u> were crafted and implemented by county and city local health jurisdictions (LHJs) to describe how resources to mitigate COVID-19 disparities would be utilized. Though these plans were submitted to the state by October 20, 2020, their efforts were implemented over time and phased in.

<u>Lastly, the vaccine equity allocation</u> was later incorporated, taking effect on March 2, 2021, to guide vaccine allocation for the general population when effective vaccines became available. Similar to the HEM, once vaccines were made available, a Vaccine Equity Metric (VEM) was also created. However, the VEM differed from the HEM in that it measured vaccines administered (instead of test positivity) at zip code tabulation areas (ZCTA) with CDPH-derived ZCTA scores (instead of census tracts) and statewide HPI quartiles (instead of county-level HPI quartiles). After prioritizing equitable vaccine distribution, the tier reopening framework thresholds were modified to include two statewide VEM goals as shown in Table B below:

Table B. Blueprint for a Safer Economy's Vaccine Equity Metric Goals.

**Doses administered in the Vaccine Equity Quartile 1 statewide**	Tier 1 Widespread (Purple)	Tier 2 Substantial (Red)	Tier 3 Moderate (Orange)	Tier 4 Minimal (Yellow)
< 2 million doses administered	Case Rate >7	Case Rate 4-7	Case Rate 1-3.9	Case Rate <1
Goal 1: 2 million doses administered	Case Rate >10	Case Rate 4-10	Case Rate 1-3.9	Case Rate <1
Goal 2: 4 million doses administered	Case Rate >10	Case Rate 6-10	Case Rate 2-5.9	Case Rate <2

Table C shows the resulting weekly tier assignments from the Tier Reopening Framework for California counties by week and whether counties were large (>106,000 county population) or small (otherwise).

Table C. Weekly Tier Assignments from the Tier Reopening Framework for Counties by Week and County Size

	Number of weeks from Jan 1, 2020	37	38	39	40	41	42	43	44	45	46	47	48	49	50	51	52	53	54	55	56	57	58	59	60	61	62	63	64	65	66	67	68	69	70	71	72	73	74	75	76	77	78	79
	Tier assignment - Day of week	Tues	Tues	Tues	Tues	Tues	Tues	Tues	Tues	Tues	Tues	Tues	Tues	Sat	Tues	Tues	Tues	Tues	Tues	Tues	Tues	Tues	Tues	Tues	Tues	Tues	Tues	Tues	Tues	Sat	Tues	Tues	Tues	Tues	Tues	Tues	Tues	Tues	Tues	Tues	Tues	Tues	Tues	Tues
	Tier Assignment - Date	8-Sep-23	15-Sep-20	22-Sep-20	29-Sep-20	6-Oct-20	13-Oct-20	20-Oct-20	27-Oct-20	3-Nov-20	10-Nov-20	17-Nov-20	24-Nov-20	28-Nov-20	1-Dec-20	8-Dec-20	15-Dec-20	22-Dec-20	29-Dec-20	5-Jan-21	12-Jan-21	19-Jan-21	26-Jan-21	2-Feb-21	9-Feb-21	16-Feb-21	23-Feb-21	2-Mar-21	9-Mar-21	13-Mar-21	16-Mar-21	23-Mar-21	30-Mar-21	6-Apr-21	13-Apr-21	20-Apr-21	27-Apr-21	4-May-21	11-May-21	18-May-21	25-May-21	1-Jun-21	8-Jun-21	15-Jun-21
Large County (>106k pop)	County																																											
1	Alameda	1	1	2	2	2	3	3	3	3	3	1	1	1	1	1	1	1	1	1	1	1	1	1	1	1	1	1	2	2	2	2	3	3	3	3	3	3	3	3	3	3	4	4
1	Butte	1	1	1	2	2	2	3	3	3	3	1	1	1	1	1	1	1	1	1	1	1	1	1	1	1	1	1	2	2	2	2	3	3	3	3	3	3	3	3	3	3	3	3
1	Contra Costa	1	1	1	2	2	2	2	3	3	2	1	1	1	1	1	1	1	1	1	1	1	1	1	1	1	1	1	1	2	2	2	2	3	3	3	3	3	3	3	3	3	3	3
1	El Dorado	2	2	3	3	3	3	3	3	3	3	1	1	1	1	1	1	1	1	1	1	1	1	1	1	1	1	2	2	2	2	2	2	3	3	3	3	3	3	3	3	3	3	3
1	Fresno	1	1	1	2	2	2	2	2	2	2	1	1	1	1	1	1	1	1	1	1	1	1	1	1	1	1	1	1	1	1	1	2	2	2	3	3	3	3	3	3	3	3	3
1	Humboldt	3	3	3	3	4	4	4	4	4	4	2	1	1	1	1	1	1	2	2	1	1	1	1	1	1	2	2	2	2	2	2	2	3	3	3	3	3	3	3	3	3	3	3
1	Imperial	1	1	1	1	1	1	1	1	1	1	1	1	1	1	1	1	1	1	1	1	1	1	1	1	1	1	1	2	2	2	2	2	3	3	3	3	3	3	3	3	3	3	3
1	Kern	1	1	1	1	1	2	2	2	2	2	1	1	1	1	1	1	1	1	1	1	1	1	1	1	1	1	1	1	1	1	2	2	2	3	3	3	3	3	3	3	3	3	3
1	Kings	1	1	1	1	1	2	2	2	2	2	1	1	1	1	1	1	1	1	1	1	1	1	1	1	1	1	1	1	1	1	1	2	2	2	3	3	3	3	3	3	3	3	3
1	Los Angeles	1	1	1	1	1	1	1	1	1	1	1	1	1	1	1	1	1	1	1	1	1	1	1	1	1	1	1	1	2	2	2	3	3	3	3	3	4	4	4	4	4	4	4
1	Madera	1	1	1	1	1	1	1	1	1	1	1	1	1	1	1	1	1	1	1	1	1	1	1	1	1	1	1	1	1	1	1	2	2	2	2	2	2	3	3	3	3	3	3
1	Marin	1	2	2	2	2	2	2	3	3	3	2	2	2	2	1	1	1	1	1	1	1	1	1	1	1	2	2	2	2	2	3	3	3	3	3	3	3	3	3	3	4	4	4
1	Merced	1	1	1	1	2	2	2	2	2	2	1	1	1	1	1	1	1	1	1	1	1	1	1	1	1	1	1	1	1	1	1	1	1	2	2	2	2	2	2	3	3	3	3
1	Monterey	1	1	1	1	1	1	1	1	1	1	1	1	1	1	1	1	1	1	1	1	1	1	1	1	1	1	1	1	1	2	2	2	3	3	3	3	3	3	3	3	4	4	4
1	Napa	2	2	2	2	2	2	3	3	3	3	1	1	1	1	1	1	1	1	1	1	1	1	1	1	1	1	2	2	2	2	2	2	3	3	3	3	3	3	3	3	3	4	4
1	Orange	2	2	2	2	2	2	2	2	2	2	1	1	1	1	1	1	1	1	1	1	1	1	1	1	1	1	1	1	2	2	2	3	3	3	3	3	3	3	4	4	4	4	4
1	Placer	2	2	2	2	2	3	3	3	3	2	1	1	1	1	1	1	1	1	1	1	1	1	1	1	1	1	1	1	2	2	2	2	2	2	2	2	2	2	2	3	3	3	3
1	Riverside	1	1	2	2	2	2	1	1	1	1	1	1	1	1	1	1	1	1	1	1	1	1	1	1	1	1	1	1	1	2	2	2	3	3	3	3	3	3	3	3	3	3	4
1	Sacramento	1	1	1	2	2	2	2	2	2	1	1	1	1	1	1	1	1	1	1	1	1	1	1	1	1	1	1	1	1	2	2	2	2	2	2	2	2	2	2	2	3	3	3
1	San Bernardino	1	1	1	1	1	1	1	1	1	1	1	1	1	1	1	1	1	1	1	1	1	1	1	1	1	1	1	1	2	2	2	2	3	3	3	3	3	3	3	3	3	3	4
1	San Diego	2	2	2	2	2	2	2	2	2	1	1	1	1	1	1	1	1	1	1	1	1	1	1	1	1	1	1	1	1	2	2	2	3	3	3	3	3	3	3	3	3	4	4
1	San Francisco	2	2	2	3	3	3	4	4	4	4	2	2	1	1	1	1	1	1	1	1	1	1	1	1	1	1	2	2	2	2	3	3	3	3	3	3	4	4	4	4	4	4	4
1	San Joaquin	1	1	1	2	2	2	2	2	2	2	1	1	1	1	1	1	1	1	1	1	1	1	1	1	1	1	1	1	1	1	1	1	2	2	2	2	2	2	2	2	3	3	3
1	San Luis Obispo	1	1	2	2	2	2	2	2	2	2	1	1	1	1	1	1	1	1	1	1	1	1	1	1	1	1	2	2	2	2	2	2	2	2	2	3	3	3	3	3	3	4	4
1	San Mateo	1	1	2	2	2	2	2	3	3	3	2	2	1	1	1	1	1	1	1	1	1	1	1	1	1	2	2	2	2	3	3	3	3	3	3	3	3	3	4	4	4	4	4
1	Santa Barbara	1	1	1	2	2	2	2	2	2	2	1	1	1	1	1	1	1	1	1	1	1	1	1	1	1	1	1	1	1	2	2	2	2	2	3	3	3	3	3	3	3	4	4
1	Santa Clara	2	2	2	2	2	3	3	3	3	3	1	1	1	1	1	1	1	1	1	1	1	1	1	1	1	1	2	2	2	2	3	3	3	3	3	3	3	3	4	4	4	4	4
1	Santa Cruz	2	2	2	2	2	2	2	3	3	2	1	1	1	1	1	1	1	1	1	1	1	1	1	1	1	1	1	2	2	2	2	3	3	3	3	3	3	3	4	4	4	4	4
1	Shasta	3	3	3	3	2	2	1	2	1	1	1	1	1	1	1	1	1	1	1	1	1	1	1	1	1	2	2	2	2	2	2	2	2	2	2	2	2	2	2	2	2	2	3
1	Solano	1	1	2	2	2	2	2	2	2	2	1	1	1	1	1	1	1	1	1	1	1	1	1	1	1	1	1	2	2	2	2	2	2	2	2	2	2	2	2	2	3	3	3
1	Sonoma	1	1	1	1	1	1	1	1	1	1	1	1	1	1	1	1	1	1	1	1	1	1	1	1	1	1	1	1	2	2	2	2	3	3	3	3	3	3	3	3	3	3	3
1	Stanislaus	1	1	1	1	1	2	2	2	2	2	1	1	1	1	1	1	1	1	1	1	1	1	1	1	1	1	1	1	1	1	2	2	2	2	2	2	2	2	2	2	2	3	3
1	Tulare	1	1	1	1	1	1	1	1	1	1	1	1	1	1	1	1	1	1	1	1	1	1	1	1	1	1	1	1	1	2	2	2	3	3	3	3	3	3	3	3	3	3	4
1	Ventura	1	1	1	1	2	2	2	2	2	2	1	1	1	1	1	1	1	1	1	1	1	1	1	1	1	1	1	1	1	2	2	2	3	3	3	3	3	3	3	3	4	4	4
1	Yolo	1	1	1	2	2	2	2	2	2	2	1	1	1	1	1	1	1	1	1	1	1	1	1	1	1	2	2	2	2	2	3	3	3	3	3	3	3	3	3	3	3	3	4
0	Mendocino	1	1	1	1	1	1	1	2	2	2	1	1	1	1	1	1	1	1	1	1	1	1	1	1	1	1	1	1	2	2	2	2	3	3	3	4	4	4	4	4	4	4	4
0	Alpine	4	4	4	4	4	4	4	4	4	4	4	3	2	2	2	2	2	2	2	2	2	2	3	3	3	3	3	4	4	4	4	4	4	4	4	4	4	4	4	4	4	4	4
0	Amador	2	2	2	3	3	3	3	3	3	2	2	2	2	2	1	1	1	1	1	1	1	1	1	1	1	1	1	1	2	2	2	2	2	2	2	3	3	3	4	4	4	4	4
0	Calaveras	2	2	2	3	3	3	3	4	4	4	3	2	1	1	1	1	1	1	1	1	1	1	1	1	1	1	1	2	2	2	2	2	2	2	3	3	3	3	3	3	3	3	3
0	Colusa	1	1	1	1	1	2	2	2	3	3	2	1	1	1	1	1	1	1	1	1	1	1	1	1	1	1	1	1	2	2	2	3	3	3	3	3	3	3	3	3	3	3	3
0	Del Norte	3	3	3	3	3	3	3	3	3	3	2	1	1	1	1	1	1	1	1	1	1	1	1	2	2	2	2	2	2	2	2	2	2	2	2	2	2	2	2	2	2	2	3
0	Glenn	1	1	1	1	1	1	1	2	2	2	1	1	1	1	1	1	1	1	1	1	1	1	1	1	1	1	1	1	1	1	1	2	2	2	2	3	3	3	3	3	3	3	4
0	Inyo	1	2	2	2	3	3	3	3	3	3	3	3	2	2	2	1	1	1	1	1	1	1	1	1	1	1	1	1	1	1	1	1	1	2	2	2	3	3	3	4	4	4	4
0	Lake	2	2	2	2	2	2	2	2	2	2	2	2	1	1	1	1	1	1	1	1	1	1	1	1	1	1	1	1	1	2	2	2	2	3	3	3	3	3	3	3	3	3	3
0	Lassen	2	2	3	3	3	3	3	3	3	3	3	1	1	1	1	1	1	1	1	1	1	1	1	1	1	1	2	2	2	2	3	3	3	4	4	4	4	4	4	4	4	4	4
0	Mariposa	3	3	4	4	4	4	4	4	4	4	4	3	2	2	2	2	2	2	2	2	2	2	2	2	2	2	2	3	3	3	3	3	3	3	3	3	3	3	3	4	4	4	4
0	Modoc	4	4	4	4	4	4	4	4	4	3	2	3	1	1	1	1	1	1	1	1	1	1	1	1	1	1	2	2	2	2	2	3	3	3	3	3	3	3	3	3	3	3	3
0	Mono	3	3	3	3	3	3	3	3	3	3	2	2	2	1	1	1	1	1	1	1	1	1	1	1	1	1	1	1	2	2	2	2	2	2	3	3	3	4	4	4	4	4	4
0	Nevada	2	2	3	3	3	3	3	3	3	3	1	1	1	1	1	1	1	1	1	1	1	1	1	1	1	1	1	1	1	1	2	2	2	2	2	2	2	2	2	2	3	3	3
0	Plumas	3	3	3	3	4	4	4	4	3	3	2	2	1	1	1	1	1	1	1	1	1	1	1	1	2	2	2	3	3	3	3	3	3	3	3	3	3	3	3	3	3	3	3
0	San Benito	1	1	1	1	1	2	2	2	2	2	1	1	1	1	1	1	1	1	1	1	1	1	1	1	1	1	1	1	2	2	2	2	3	3	3	3	3	3	3	3	4	4	4
0	Sierra	2	2	3	3	3	4	4	4	4	4	3	3	3	3	3	3	3	3	3	3	3	3	3	3	3	3	3	3	3	3	4	4	4	4	4	4	4	4	4	4	4	4	4
0	Siskiyou	3	3	3	3	4	4	4	4	4	3	1	1	1	1	1	1	1	1	1	1	1	1	1	1	1	1	1	1	2	2	2	2	3	3	3	3	3	3	3	3	3	3	3
0	Sutter	1	1	1	1	1	2	2	2	2	2	1	1	1	1	1	1	1	1	1	1	1	1	1	1	1	1	1	1	1	2	2	2	2	2	2	3	3	3	3	3	3	3	3
0	Tehama	1	2	2	2	1	1	1	1	1	1	1	1	1	1	1	1	1	1	1	1	1	1	1	1	1	1	1	1	1	2	2	2	2	2	2	2	2	2	3	3	3	3	3
0	Trinity	3	3	3	3	4	4	4	4	4	3	1	1	1	1	1	1	1	1	1	2	2	2	3	3	3	2	2	2	2	2	3	3	3	3	3	3	4	4	4	4	4	4	4
0	Tuolumne	3	3	3	3	3	3	3	3	3	3	1	1	1	1	1	1	1	1	1	1	1	1	1	1	1	1	1	1	2	2	2	3	3	3	3	3	3	3	3	3	3	3	3
0	Yuba	1	1	1	1	2	2	2	2	2	2	1	1	1	1	1	1	1	1	1	1	1	1	1	1	1	1	1	1	1	1	1	2	2	2	2	2	2	2	2	2	2	2	2

Note: Large (versus small) county designation is based on population ACS 2015-2019 5-year estimates for population. Large counties based on Blueprint criteria were those with 106,000 residents or more, and small counties were those with fewer than 106,000 residents. Data is from the California Health and Human Services Open Data Portal.
